# Structure and dynamics of SARS-CoV-2 proofreading exoribonuclease ExoN

**DOI:** 10.1073/pnas.2106379119

**Published:** 2022-02-14

**Authors:** Nicholas H. Moeller, Ke Shi, Özlem Demir, Christopher Belica, Surajit Banerjee, Lulu Yin, Cameron Durfee, Rommie E. Amaro, Hideki Aihara

**Affiliations:** ^a^Department of Biochemistry, Molecular Biology and Biophysics, University of Minnesota, Minneapolis, MN 55455;; ^b^Institute for Molecular Virology, University of Minnesota, Minneapolis, MN 55455;; ^c^Masonic Cancer Center, University of Minnesota, Minneapolis, MN 55455;; ^d^Department of Chemistry and Biochemistry, University of California San Diego, La Jolla, CA 92093;; ^e^Northeastern Collaborative Access Team, Advanced Photon Source, Cornell University, Lemont, IL 60439

**Keywords:** SARS-CoV-2, exoribonuclease, proofreading, molecular dynamics simulations, crystal structure

## Abstract

High-fidelity replication of the large RNA genome of coronaviruses (CoVs) is mediated by a 3′-to-5′ exoribonuclease (ExoN) in nonstructural protein 14 (nsp14), which excises nucleotides including antiviral drugs misincorporated by the low-fidelity viral RNA-dependent RNA polymerase (RdRp) and has also been implicated in viral RNA recombination and resistance to innate immunity. Here, we determined a 1.6-Å resolution crystal structure of severe acute respiratory syndrome CoV 2 (SARS-CoV-2) ExoN in complex with its essential cofactor, nsp10. The structure shows a highly basic and concave surface flanking the active site, comprising several Lys residues of nsp14 and the N-terminal amino group of nsp10. Modeling suggests that this basic patch binds to the template strand of double-stranded RNA substrates to position the 3′ end of the nascent strand in the ExoN active site, which is corroborated by mutational and computational analyses. We also show that the ExoN activity can rescue a stalled RNA primer poisoned with sofosbuvir and allow RdRp to continue its extension in the presence of the chain-terminating drug, biochemically recapitulating proofreading in SARS-CoV-2 replication. Molecular dynamics simulations further show remarkable flexibility of multidomain nsp14 and suggest that nsp10 stabilizes ExoN for substrate RNA binding to support its exonuclease activity. Our high-resolution structure of the SARS-CoV-2 ExoN–nsp10 complex serves as a platform for future development of anticoronaviral drugs or strategies to attenuate the viral virulence.

## Significance

SARS-CoV-2 nonstructural protein 14 (nsp14) exoribonuclease (ExoN) plays important roles in the proofreading during viral RNA synthesis and the evasion of host immune responses. We used X-ray crystallography, molecular dynamics simulations, and biochemical assays to investigate the structure, dynamics, and RNA-binding mechanisms of nsp14-ExoN and how its activity is regulated by another viral protein, nsp10. We also demonstrated that nsp14-ExoN can collaborate with the viral RNA polymerase to enable RNA synthesis in the presence of a chain-terminating drug, biochemically recapitulating the proofreading process. Our studies provide mechanistic insights into the functions of a key viral enzyme and a basis for future development of chemical inhibitors.

The 29.9-kb single-stranded RNA genome of severe acute respiratory syndrome coronavirus 2 (SARS-CoV-2), the causative agent of the global COVID-19 pandemic, is replicated and transcribed by the viral RNA-dependent RNA polymerase (RdRp, nsp12) (1–3). Unlike the high-fidelity cellular replicative DNA polymerases, viral RdRp enzymes, including the CoV RdRp, do not contain a proofreading exonuclease domain to ensure high fidelity. The resulting higher mutation rate (10^−4^ to 10^−6^ substitutions per nucleotide per round of replication) is generally thought to promote rapid viral adaptation in response to selective pressure (4–6). However, the lack of proofreading activity in RdRp poses a particular challenge for the replication of CoVs, which feature the largest known RNA virus genomes (27 to 32 kb, up to twice the length as the next-largest nonsegmented RNA viral genomes) (7, 8). It has been reported that SARS-CoV nsp12 is the fastest viral RdRp known but with an error rate more than one order of magnitude higher than the generally admitted error rate of viral RdRps (9), clearly necessitating a unique proofreading mechanism.

To mitigate the low fidelity of RdRp, all coronaviruses encode a 3′-to-5′ exoribonuclease (ExoN) in multifunctional nsp14 (10–12), which forms a complex with nsp10 critical for the ExoN activity, and additionally contains a C-terminal guanine N7 methyl transferase (N7-MTase) domain. Mutations of SARS-CoV-2 nsp14 exhibit strong association with increased genome-wide mutation load (13, 14), and genetic inactivation of ExoN in engineered SARS-CoV and murine hepatitis virus (MHV) leads to 15- to 20-fold increases in mutation rates (7, 15, 16). Furthermore, in a mouse model, SARS-CoV with inactivated ExoN shows a mutator phenotype with decreased fitness and lower virulence over serial passage, suggesting a potential strategy for generating a live, impaired-fidelity coronavirus vaccine (17). Alternatively, recent studies show that ExoN inactivation abrogates replication of SARS-CoV-2 and Middle East Respiratory Syndrome CoV (18), hinting at additional functions for ExoN in viral replication. Indeed, ExoN activity has been reported to mediate the extensive viral RNA recombination required for subgenomic messenger RNA (mRNA) synthesis during normal replication of CoVs, including SARS-CoV-2 (19), and it was shown to be required for resistance to the antiviral innate immune response for MHV (20). ExoN inactivation also significantly increases the sensitivity of CoVs to nucleoside analogs that target RdRp, which is consistent with the biochemical activity of ExoN to excise mutagenic or chain-terminating nucleotides misincorporated by RdRp (21–23). These observations combine to suggest that chemical inhibition of ExoN could be an effective antiviral strategy against CoVs. In this study, we determined high-resolution crystal structures of the SARS-CoV-2 ExoN–nsp10 complex and studied its biochemical activities. Furthermore, we used molecular dynamics (MD) simulations to better understand the dynamics of nsp14, nsp10, and their interaction with RNA.

## Results

**Fig. 1.**
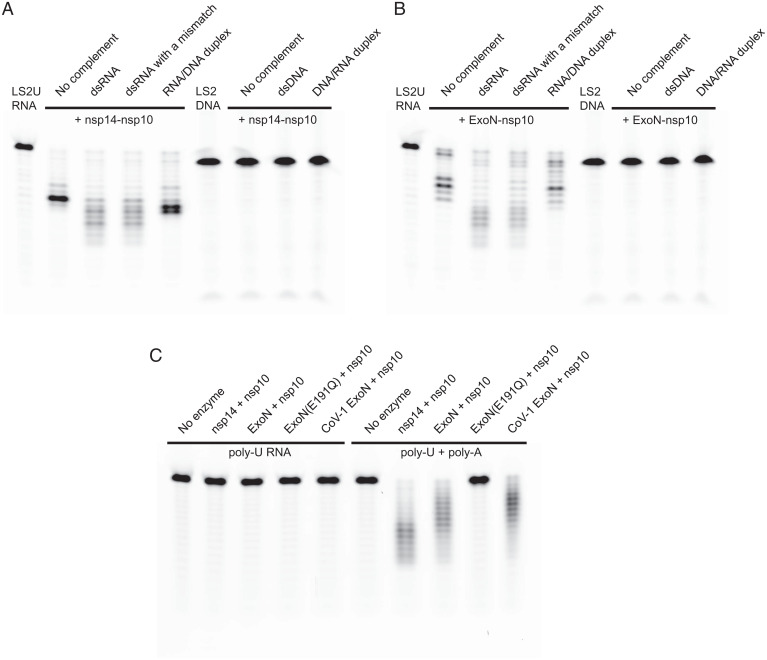
Biochemical activities of nsp14 or its N-terminal ExoN domain, in complex with nsp10. (*A*) Exonuclease activities of SARS-CoV-2 full-length nsp14–nsp10 complex on various RNA and DNA substrates. (*B*) Exonuclease activities of SARS-CoV-2 ExoN (nsp14 residues 1 to 289)–nsp10 complex on the same set of RNA and DNA substrates as in *A*. (*C*) Exonuclease activities of SARS-CoV-2 full-length nsp14–nsp10, SARS-CoV-2 ExoN–nsp10, and SARS-CoV ExoN–nsp10 complexes on poly-U RNA in the absence (*Left*) or presence (*Right*) of unlabeled poly-A RNA. Please see Table 1 for the substrate sequences.

The multifunctional SARS-CoV-2 nsp14 consists of the N-terminal ExoN domain involved in proofreading and the C-terminal N7-MTase domain that functions in mRNA capping. We coexpressed, in bacteria, the full-length 527-residue SARS-CoV-2 nsp14 or its N-terminal fragment (residues 1 to 289) containing only the ExoN domain, with full-length 139-residue nsp10 in both cases, and purified the heterodimeric complexes. The nsp14–nsp10 and ExoN–nsp10 complexes both showed the expected 3′-to-5′ exonuclease activity on a 5′ fluorescently labeled 20-nucleotide (nt) RNA [LS2U: 5′-GUCAUUCUCCUAAGAAGCUU; similar to “LS2” used previously in SARS-CoV ExoN studies (21)] (Fig. 1 *A* and *B*). Although LS2U RNA by itself served as a substrate, more extensive degradation was observed when it was annealed to an unlabeled 40-nt template strand (LS15A_RNA; Table 1) to generate a double-stranded (ds)RNA with a 20-nt 5′ overhang. Introducing a base mismatch at the 3′ end of the degradable strand by using an alternative bottom strand (LS15_RNA; Table 1) had no discernable effect on the processing by either complex (Fig. 1 *A* and *B*). When DNA was used as the template strand (LS15_DNA; Table 1) to generate an RNA/DNA heteroduplex substrate that is expected to take the A-form conformation similarly to dsRNA, the activity was observed but was weaker than for dsRNA. No nuclease activity was observed on a 5′ fluorescently labeled 20-nt DNA (LS2_DNA; Table 1), whether the template strand was RNA (LS15_RNA), DNA (LS15_DNA; Table 1), or absent. A 20-nt poly-U RNA (U20_RNA; Table 1), which is less likely to adopt secondary structures than LS2U, did not serve as a substrate by itself but was degraded extensively when supplemented with a complementary 30-nt poly-A RNA (A30_RNA; Table 1) (Fig. 1*C*). Collectively, these results show that the N-terminal ExoN domain of SARS-CoV-2 nsp14 is sufficient for binding to nsp10 to form an active exonuclease complex that preferentially degrades dsRNA. For comparison, we also generated a corresponding SARS-CoV ExoN–nsp10 complex, which showed activities similar to SARS-CoV-2 ExoN–nsp10 (Fig. 1*C* and *SI Appendix*, Fig. S1).

**Table 1.** Oligonucleotides used in biochemical assays

Name	Sequence
LS2U	/56-FAM/rGrUrCrArUrUrCrUrCrCrUrArArGrArArGrCrUr**U**
LS2U-d	/56-FAM/rGrUrCrArUrUrCrUrCrCrUrArArGrArArGrCrU/3deoxyU/
LS2U-F	/56-FAM/rGrUrCrArUrUrCrUrCrCrUrArArGrArArGrCrU/32FU/
LS2U-ddd	/56-FAM/rGrUrCrArUrUrCrUrCrCrUrArArGrArArGC/ideoxyU//3deoxyU/
LS2U-FFF	/56-FAM/rGrUrCrArUrUrCrUrCrCrUrArArGrArArG/i2FC//i2FU//32FU/
LS15A_RNA	rCrUrArUrCrCrCrCrArUrGrUrGrArUrUrUrUrArCr**A**rArGrCrUrUrCrUrUrArGrGrArGrArArUrGrArC
LS15_RNA	rCrUrArUrCrCrCrCrArUrGrUrGrArUrUrUrUrArCr**U**rArGrCrUrUrCrUrUrArGrGrArGrArArUrGrArC
LS2_DNA	/56-FAM/GTCATTCTCCTAAGAAGCTA
LS15_DNA	CTATCCCCATGTGATTTTACTAGCTTCTTAGGAGAATGAC
U20_RNA	/56-FAM/rUrUrUrUrUrUrUrUrUrUrUrUrUrUrUrUrUrUrUrU
A30_RNA	rArArArArArArArArArArArArArArArArArArArArArArArArArArArArArA

Notation is as follows: 56-FAM: 5′ 6-fluorescein; r: ribonucleotide; deoxyU: 2′-deoxyuridine; 2FU: 2′-fluorouridine; 3′ indicates 3′ end of an oligonucleotide, whereas i indicates internal modification.

**Fig. 2.**
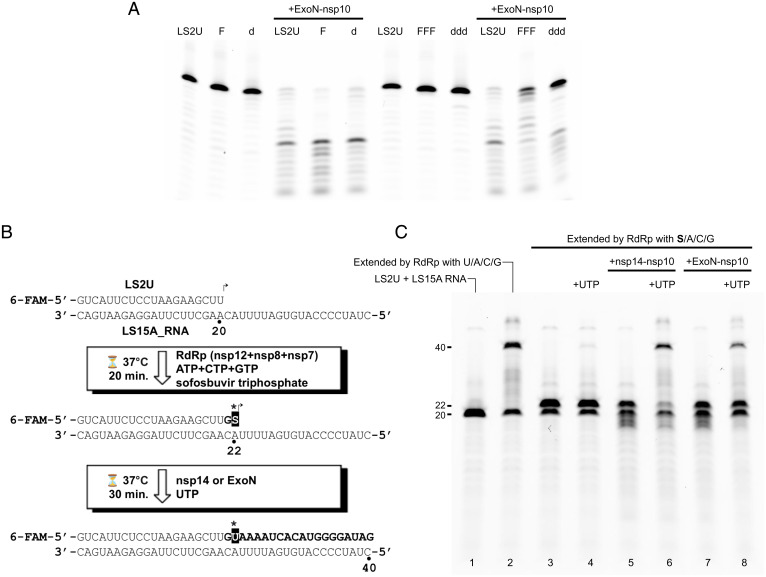
ExoN activities on modified RNA substrates and a chain-terminating drug sofosbuvir. (*A*) Exonuclease activities of SARS-CoV-2 ExoN (nsp14 residues 1 to 289)–nsp10 complex on RNA substrates with modifications on the 3′-terminal nucleotide (LS2U-F: 2′-fluoro; LS2U-d: 2′-deoxy) or three 3′-terminal nucleotides (LS2U-FFF: 2′-fluoro; LS2U-ddd: 2′-deoxy). LS2U is unmodified RNA. All substrates were annealed with the fully complementary LS15A RNA. (*B*) Schematic of the “sofosbuvir rescue” experiment, result of which is shown in *C*. (*C*) Extension of sofosbuvir-terminated RNA primer by RdRp in the presence of nsp14–nsp10 or ExoN–nsp10 complex. Lane 1: unextended primer. Lane 2: primer extended with natural NTPs. Lane 3: primer extended with ATP, CTP, GTP, and sofosbuvir triphosphate, which served as the starting material for lanes 4 to 8. Lanes 4 to 6: after 30-min incubation with UTP (lane 4), nsp14–nsp10 (lane 5), or both UTP and nsp14–nsp10 (lane 6) added. Lanes 7 and 8: after 30-min incubation with ExoN–nsp10 (lane 7) or both UTP and ExoN–nsp10 (lane 8) added.

To better understand the basis of the strong preference for RNA over DNA substrate above, we further tested the activity of SARS-CoV-2 ExoN–nsp10 on dsRNA substrates containing 2′-deoxy or 2′-fluoro substitutions. A 2′-fluoro substitution sterically mimics the 2′-hydroxyl group in RNA but removes its hydrogen-bonding capacity. When introduced at the single 3′-terminal nucleotide of the degradable strand, neither 2′-deoxy nor 2′-fluoro substitution (LS2U-d/LS2U-F; Table 1) had any discernible effect (Fig. 2 *A*, *Left*). On the other hand, three consecutive 2′-deoxy substitutions at the 3′ end (LS2U-ddd; Table 1) strongly inhibited degradation by ExoN–nsp10, whereas three consecutive 2′-fluoro modifications at the 3′ end (LS2U-FFF; Table 1) modestly inhibited the degradation (Fig. 2 *A*, *Right*). These results suggest that both the conformation and hydrogen-bonding potential of the second and/or third nucleotides from the 3′ end are important for substrate recognition. We also examined whether the ExoN activity can remove remdesivir, an antiviral drug Food and Drug Administration–approved for COVID-19 treatments, misincorporated by RdRp. SARS-CoV-2 RdRp (nsp12/nsp7/nsp8) readily extended the LS2U primer annealed to the LS15A RNA template into a 40-nt product using remdesivir triphosphate in place of adenosine 5′-triphosphate (ATP), additionally generating small fractions of internally chain-terminated products as confirmed by mass spectrometry (*SI Appendix*, Fig. S2). We found that both nsp14–nsp10 and ExoN–nsp10 complexes can degrade the full-length and abortive extension products containing multiple remdesivir incorporations, similarly to how they process the extension product obtained only using natural ribonucleotides (*SI Appendix*, Fig. S2).

An anti-hepatitis C virus drug and a stronger chain terminator, sofosbuvir, has also been shown to have antiviral activity against SARS-CoV-2 in vitro (24), and it was reported to be more resistant to removal by SARS-CoV-2 nsp14 than remdesivir (25). Thus, we next sought to biochemically recapitulate proofreading in SARS-CoV-2 replication and tested whether the ExoN activity can rescue an RNA primer stalled with sofosbuvir to allow RdRp to resume extension (Fig. 2 *B* and *C*). The LS2U primer annealed to the LS15A RNA template was first extended by RdRp in the presence of ATP, guanosine 5′-triphosphate (GTP), cytidine 5′-triphosphate (CTP), and sofosbuvir triphosphate in place of uridine 5′-triphosphate (UTP), which causes stalling of the primer extension after incorporation of two nucleotides. Subsequent addition of UTP alone allowed generation of little full-length (40-nt) extension product, suggesting that a breakthrough extension or the extension of initially unused primer is negligible. In contrast, addition of the nsp14–nsp10 complex along with UTP led to removal of sofosbuvir from the stalled primers and the subsequent extension to full-length RNA products in the presence of sofosbuvir triphosphate (Fig. 2*C*). A similar result was obtained with the ExoN–nsp10 complex, albeit requiring a higher enzyme concentration. These results demonstrate that nsp14/ExoN can cooperate with RdRp to facilitate faithful viral genome replication in the presence of chain-terminating drugs.

**Fig. 3.**
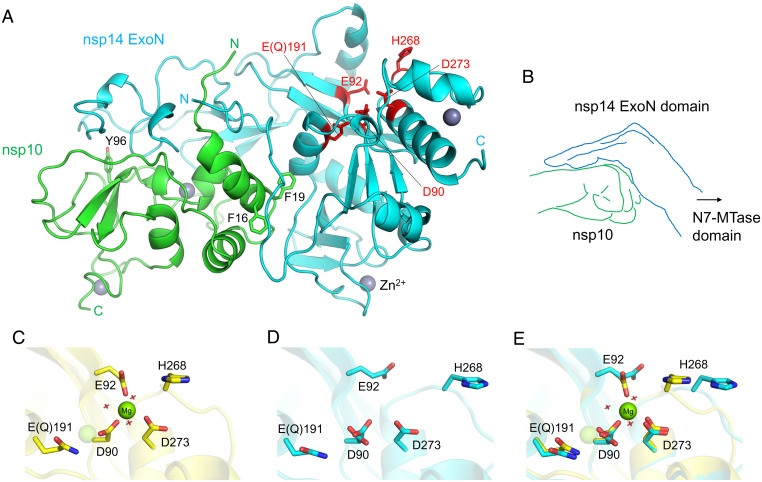
SARS-CoV-2 ExoN–nsp10 structure and its active site flexibility. (*A*) Overall structure of nsp14(1 to 289)–nsp10 complex. The N-terminal ExoN domain of nsp14 is shown in cyan, and nsp10 is shown in green. The ExoN active site residues are highlighted as red sticks. Key aromatic residues of nsp10 in the protein–protein interface are also shown as sticks. Gray spheres represent zinc ions. (*B*) A schematic illustration of hand (ExoN) over a fist (nsp10). (*C*) ExoN active site in the presence of Mg^2+^. The magnesium ion is shown as a solid sphere scaled at half the van der Waals radius. The second Mg^2+^-binding site, indicated by a transparent sphere, is unoccupied in our structure, presumably due to the E191Q mutation. Red crosshairs indicate water molecules. (*D*) Mg^2+^-free active site as observed in the tartrate-bound crystal. Asp90 side chain shows a dual conformation. (*E*) Superposition of *C* and *D* highlighting the conformational changes upon Mg^2+^ binding.

Previous X-ray crystallographic studies have provided the structure of SARS-CoV nsp14–nsp10 complex at resolutions ranging from 3.2 Å to 3.4 Å (21, 26). To obtain a higher-resolution view of a CoV ExoN complex and to reveal possible structural differences between SARS-CoV and SARS-CoV-2 ExoN, we have crystallized the SARS-CoV-2 ExoN–nsp10 complex. An ExoN variant with a nuclease-inactivating mutation (E191Q) (Fig. 1*C* and *SI Appendix*, Fig. S1) was used in our crystallographic studies, as it was expressed more robustly and generated a more stable complex with nsp10 than wild-type ExoN. We obtained crystals under two different conditions, one containing ammonium tartrate and the other containing magnesium chloride (MgCl_2_), albeit in the same crystal form. The structures were determined by molecular replacement phasing and refined to 1.64- and 2.10-Å resolution for the tartrate and magnesium-bound crystals, respectively (Fig. 3*A* and Table 2). The final models consist of nsp14 residues Asn3 to Arg289 (Val287 for the lower-resolution structure) and nsp10 residues Ala1 to Cys130, with two zinc ions bound to each polypeptide chain. As expected from high sequence conservations, SARS-CoV-2 ExoN–nsp10 complex shows high structural similarity to its counterpart from SARS-CoV [rmsd of 0.95 Å for all main chain atoms against Protein Data Bank (PDB) entry 5C8T (26)], whose shape was previously described to resemble “hand (ExoN) over a fist (nsp10)” (21) (Fig. 3*B*). A superposition between the SARS-CoV and SARS-CoV-2 ExoN–nsp10 structures shows only relatively small (3.0 Å or less) deviations in several regions of the complex, including the tip of the “fingers” region of ExoN comprising nsp14 residues 40 to 50, and surface-exposed loops of nsp10 (*SI Appendix*, Fig. S3).

**Table 2.** Summary of X-ray data collection and model refinement statistics

	ExoN–nsp10 (7MC5)	ExoN–nsp10–Mg^2+^ (7MC6)
Data collection		
Wavelength (Å)	0.979	0.979
Resolution range (Å)	57.7–1.64 (1.70–1.64)	42.6–2.10 (2.18–2.10)
Space group	*P*2_1_2_1_2_1_	*P*2_1_2_1_2_1_
Unit cell (*a*,*b*,*c* in Å)	63.74 67.48 111.25	61.67 70.32 108.54
Total reflections	258,196 (22,096)	105,896 (10,815)
Unique reflections	58,702 (5,273)	27,756 (2,767)
Multiplicity	4.4 (4.2)	3.8 (3.9)
Completeness (%)	98.81 (90.43)	98.25 (99.43)
* *<*I/*σ(*I*)>	12.57 (1.48)	10.70 (1.96)
R_merge_	0.148 (1.22)	0.078 (0.928)
R_meas_	0.166 (1.40)	0.091 (1.082)
R_p.i.m._	0.076 (0.660)	0.045 (0.543)
CC_1/2_	0.995 (0.394)	0.997 (0.524)
Refinement		
Reflections, working set	58,626 (5,273)	27,755 (2,768)
Reflections, test set	2,826 (251)	1,364 (132)
* R*_work_	0.166 (0.354)	0.197 (0.306)
* R*_free_	0.197 (0.371)	0.219 (0.346)
No. of non-H atoms	3,890	3,447
Macromolecules	3,264	3,221
Ligands	117	42
Solvent	509	184
Protein residues	417	415
rms deviations		
Bond length (Å)	0.011	0.001
Bond angles (deg)	1.10	0.41
Ramachandran plot		
Favored (%)	96.85	96.84
Allowed (%)	2.91	2.92
Outliers (%)	0.24	0.24
Average *B* factor (Å^2^)	26.61	44.43
Macromolecules	24.60	44.11
Ligands	37.61	54.76
Solvent	36.94	47.72

Statistics for the highest-resolution shell are shown in parentheses.

While our structures of SARS-CoV-2 ExoN–nsp10 obtained in the two different crystallization conditions are highly similar, they show notable differences in the exonuclease active site located around the “knuckles” of ExoN. In the crystal grown in the presence of MgCl_2_, we observed a magnesium ion octahedrally coordinated by Asp90, Glu92, Asp273, and three water molecules (Fig. 3*C* and *SI Appendix*, Fig. S4*D*). Another magnesium ion required for the conserved two-metal ion mechanism of 3′-5′ editing exonucleases (27, 28) was not observed. The previously reported SARS-CoV nsp14–nsp10 structures also showed only one metal ion, bound at an alternative site between Asp90 and Glu191 (21, 26). This site is unoccupied in our structure, presumably due to the E191Q mutation. In contrast, the higher-resolution tartrate-bound structure shows a unique configuration of the metal-free active site (Fig. 3*D* and *SI Appendix*, Fig. S4*C*). Without the magnesium ion, Asp90 takes two distinct conformers with its carboxylate group in orthogonal orientations. Glu92 is pointed away from Asp90/Asp273 and hydrogen bonded to Gln108 side chain, whereas His268, in turn, is flipped away from Glu92. A comparison between the Mg^2+^-bound and free structures shows a significant rearrangement for residues Gly265 to Val269 including the main chain atoms, accompanying an inward movement of His268 upon Mg^2+^ binding (Fig. 3*E*). These observations demonstrate high flexibility of the ExoN active site in the absence of divalent metal cofactors.

**Fig. 4.**
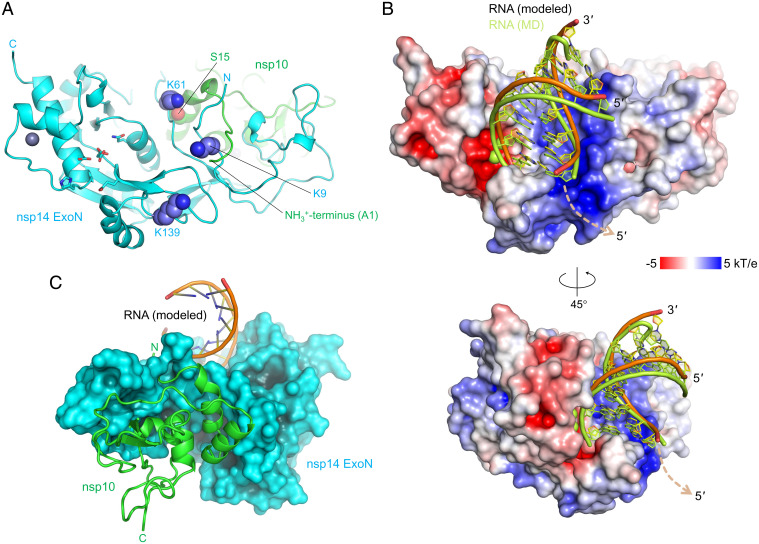
Location of the basic patch and an ExoN–nsp10-RNA complex model. (*A*) Locations of the ExoN lysine residues forming the basic patch, shown as spheres. Note that Lys9 and Lys61 interact with the N terminus (Ala1) and Ser15 of nsp10, respectively. ExoN active site residues are shown as sticks, as in Fig. 3*D*. (*B*) A hypothetical model of ExoN–nsp10-dsRNA complex, viewed from two different orientations. The protein surface is colored according to the electrostatic potential calculated using APBS (52). The RNA molecule before and after MD simulations is shown. A possible path for the single-stranded RNA continuation (5′ overhang) of the template strand is indicated by a dashed arrow. The orientation in the *Top* is the same as that in *A*. (*C*) Backside of the ExoN–nsp10–dsRNA model, viewed from the ExoN–nsp10 interface. Nsp10 is shown as green ribbon.

To obtain an idea about how SARS-CoV-2 ExoN–nsp10 complex engages RNA substrates, we modeled an RNA-bound ExoN–nsp10 structure based on the dsRNA-bound structures of Lassa virus nucleoprotein (NP) exonuclease domain, which is another DEDDh-family 3′-to-5′ RNA exonuclease important in immune evasion (29). A superposition of the Lassa NP–RNA complex (30, 31) on ExoN–nsp10 based on their conserved catalytic residues [Lassa NP: D389/E391/D466/H528/D533 according to the numbering in PDB entry 4FVU (30), vs. SARS-CoV-2 ExoN: D90/E92/E191/H268/D273] places the A-form dsRNA in a shallow groove on the ExoN surface adjacent to the active site, with remarkable shape complementarity (Fig. 4 *B* and *C*). In this model, the sugar phosphate backbone of the nondegradable (template) RNA strand tracks a positively charged patch on the ExoN surface including Lys9 and Lys61, whereas the 3′ end of its complementary (degradable) strand is presented to the active site. The extensive protein contacts made by the nondegradable strand in a dsRNA substrate is consistent with the preference for dsRNA substrates by SARS-CoV-2 ExoN as shown above (Fig. 1) and by SARS-CoV ExoN reported earlier (32). Notably, we observed ordered tartrate ions from the crystallization condition bound to this basic patch in our crystal structure, potentially mimicking RNA backbone phosphate interactions (*SI Appendix*, Figs. S4 *A* and *B* and S5).

**Fig. 5.**
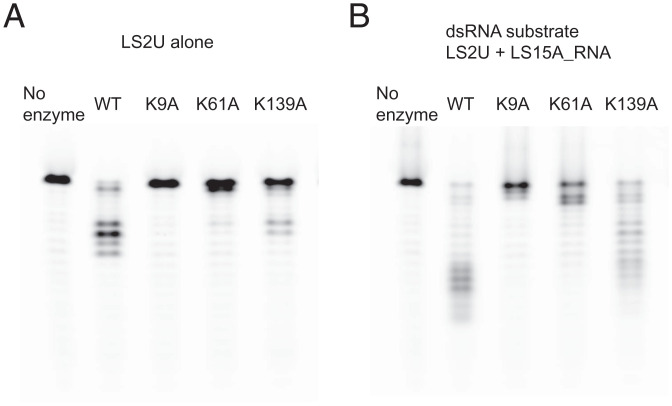
Exonuclease activities of SARS-CoV-2 ExoN–nsp10 complex and its lysine-to-alanine point mutant derivatives. (*A*) Processing of LS2U RNA without a complementary strand. (*B*) LS2U RNA annealed with the fully complementary LS15A RNA (dsRNA substrate). Please see Table 1 for the substrate sequences.

Our hypothetical model described above suggests that the basic patch of ExoN helps position the substrate RNA for exonucleolytic degradation. Lys9 and Lys61 are involved in the RNA backbone interaction in our model. In addition, Lys139 is located farther down along the basic patch toward the direction of the 5′ overhang of the template strand (Fig. 4*A*). Thus, we tested the activities of SARS-CoV-2 ExoN with single amino acid substitutions, K9A, K61A, and K139A. These ExoN mutants were coexpressed with nsp10 and purified as heterodimeric complexes. In the exonuclease assay using the RNA substrates described above, all three lysine-to-alanine mutants showed lower activity than wild-type ExoN (Fig. 5). In particular, the K9A and K61A substitutions caused severer defects than K139A, consistent with our dsRNA-binding model (Fig. 4 *B* and *C*). While the precise conformation of LS2U RNA in the absence of a complementary strand is unknown, its binding to ExoN must also depend on these Lys residues, underscoring the importance of electrostatic interactions with RNA by the mutated lysine residues in the ExoN activity. Of note, while this paper was in revision, cryoelectron microscopy (cryo-EM) structures of a SARS-CoV-2 nsp14–nsp10–dsRNA complex were reported (33). A superposition of the cryo-EM structure with our model (*SI Appendix*, Fig. S6) shows an overall similar positioning, except that dsRNA is bound slightly more shallowly in the basic groove and the last base pair is disrupted in the cryo-EM structure, which used a mismatch-containing dsRNA substrate. Due to the slightly different backbone path, Lys61 is ∼10 Å away from the closest RNA phosphate group in the cryo-EM structure. Thus, the defect caused by the K61A amino acid substitution in our experiments could represent an indirect effect, or, alternatively, ExoN may have some flexibility in RNA binding, especially for nucleotides distal to the active site.

Previous studies showed that the ExoN activity of nsp14 is strongly stimulated by nsp10 for both SARS-CoV and SARS-CoV-2 (32, 34–36). In our crystal structure, the N-terminal residues of ExoN and those of nsp10 are wrapped around each other in a “criss-cross” arrangement and forming several hydrogen bond contacts, including one between nsp14 Lys9 and nsp10 Ala1 (*SI Appendix*, Fig. S4*A*). In addition, the first α-helix of nsp10 interacts with the ExoN loop harboring nsp14 Lys61, where the main chain amide group of Lys61 is hydrogen bonded to the side chain of nsp10 Ser15 (Fig. 4*A*). In the absence of nsp10 supporting the RNA-binding groove from the back (Fig. 4*C* and *SI Appendix*, Figs. S6 and S7), the N-terminal residues of ExoN including nsp14 Lys9 and the loop residues around Lys61 are likely to be more flexible. Moreover, the terminal amino group of nsp10 Ala1 is part of the basic patch and involved in direct RNA backbone contact in our protein–RNA docking model (Fig. 4*B* and *SI Appendix*, Fig. S6). These observations may together explain the strong stimulation of ExoN activity by nsp10.

**Fig. 6.**
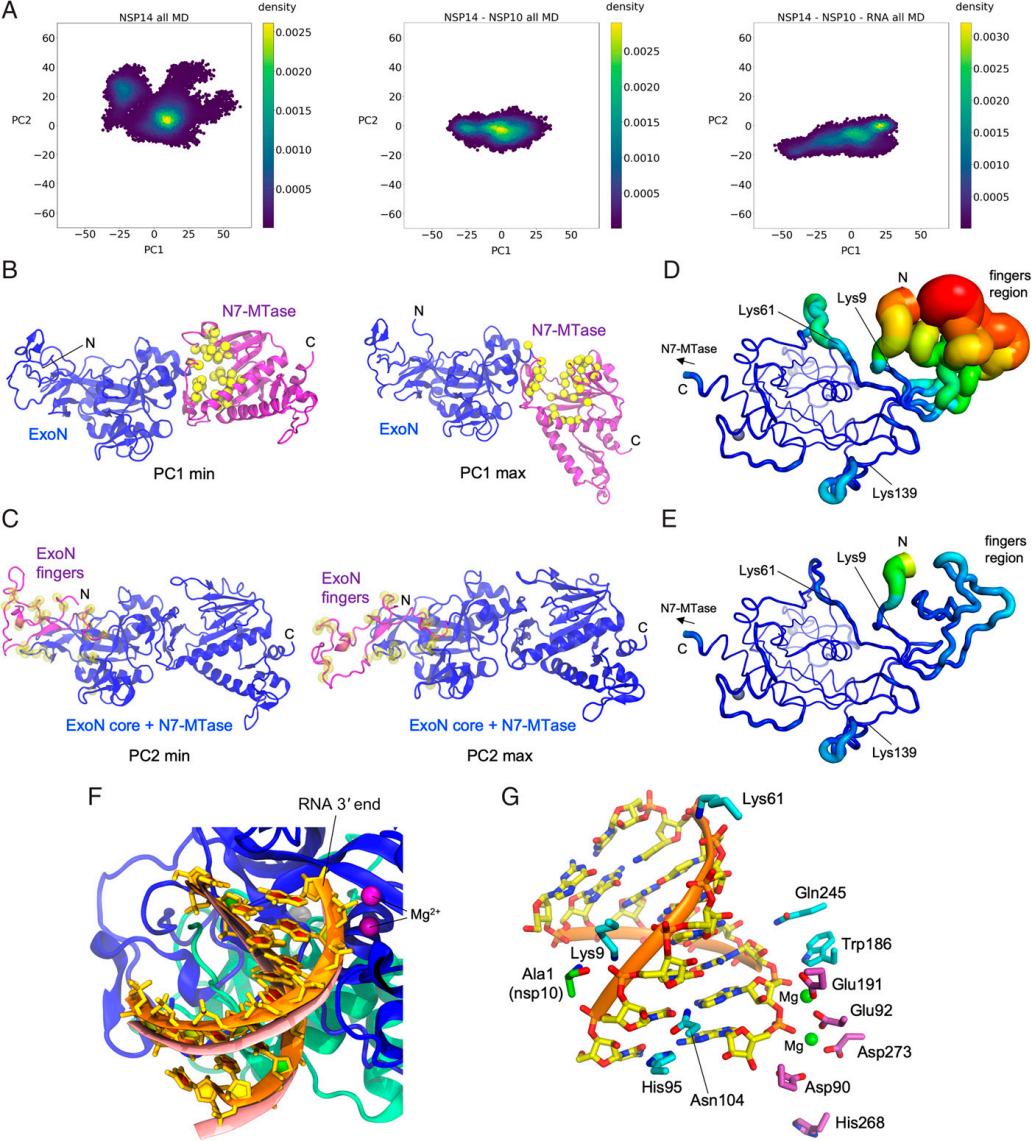
MD simulations. (*A*) Principal component analysis depicting differential conformational sampling for the three systems in MD simulations. (*B*) Structures that correspond to PC1 minimum and maximum values for the nsp14-alone system. N7-MTase and ExoN domains of nsp14 are depicted in purple and blue ribbons, respectively. Yellow spheres represent the Cα atoms of residues that constitute the binding site of SAM and GpppA substrates of N7-MTase based on homology to SARS-CoV nsp14 N7-MTase crystal structures (PDB IDs: 5C8S and 5C8T) (26). (*C*) Structures that correspond to PC2 minimum and maximum values for the nsp14-alone system. N-terminal region (residues 1 to 71) of nsp14 is depicted in purple ribbons, while the rest of nsp14 is depicted in blue ribbons. Transparent yellow spheres represent the Cα atoms of nsp14 residues that constitute the nsp10 binding site. (*D*) ExoN domain in nsp14-alone system with rms fluctuations (RMSF) of Cα atoms depicted on the structure with varying tube thickness and color (low in blue to high in red). The view is similar to that in Fig. 4*A*. (*E*) ExoN domain of nsp14–nsp10 system with Cα RMSF depicted on the structure with varying tube thickness and color. (*F*) RNA after 1-μs MD simulation (in orange ribbons) of nsp14–nsp10–RNA system superimposed onto RNA of the starting model (salmon). Nsp14 and nsp10 are depicted as blue and green ribbons, respectively. Dark purple spheres represent two Mg ions in the active site. (*G*) RNA after 1-μs MD simulation of the nsp14–nsp10–RNA system, with nsp14 ExoN domain (cyan) or nsp10 (green) residues making persistent hydrogen bond or salt bridge interactions with RNA in MD simulations shown as sticks. The active site residues of ExoN are also shown (purple sticks), with two Mg^2+^ ions as green spheres.

To obtain further insights into the role of nsp10 and to support our RNA-binding model, we performed explicitly solvated, all-atom MD simulations of full-length SARS-CoV-2 nsp14, constructed from our ExoN–nsp10 cocrystal structure and a homology model of the C-terminal N7-MTase domain. Three independent copies of MD simulations totaling 2.6 μs were performed for each of nsp14 alone, nsp14–nsp10 complex, and the nsp14–nsp10–RNA complex based on our docking model described above. In addition, three independent copies of Gaussian-accelerated MD simulations (GAMD) totaling 0.6 μs were performed for each system to enhance conformational sampling. Comparing trajectories of these simulations for the three systems, the most noticeable difference is an extreme flexibility of the “fingers” region of ExoN primarily comprising its N-terminal residues (nsp14 residues 1 to 60), which showed large deviations from the starting model and eventually became highly disordered in the absence of nsp10. A principal component analysis for the three systems shows that the conformational space sampled by nsp14 is significantly larger in the absence of nsp10 (Fig. 6*A* and *SI Appendix*, Fig. S8).

The first principal component (PC1), which is broadly sampled by all three systems, corresponds to a large hinge motion of the N7-MTase domain (∼50-Å translocation at the distal end; *SI Appendix*, Fig. S9 and Movie S1). In the conformation with minimal PC1 (Fig. 6 *B*, *Left*), the substrates (S-adenosyl methionine [SAM] and GpppA)-binding cleft of the N7-MTase domain abuts against the ExoN domain, leading to occlusion of the substrates. On the other extreme with maximal PC1, the cleft is more open to the solvent (Fig. 6 *B*, *Right*). The second principal component (PC2) corresponds to an ordered-to-disordered transition of the “fingers” region of ExoN, which shows a large population of disordered conformations only for the nsp14-alone system as mentioned above (Fig. 6*C* and *SI Appendix*, Fig. S10 and Movies S2 and S3). Although folding of the core of the ExoN domain does not depend on nsp10, residues Lys9 and Lys61 important for RNA binding and the surrounding residues show increased flexibility in the absence of nsp10, confirming our prediction above (Table 3, Fig. 6 *D* and *E*, *SI Appendix*, Fig. S10, and Movie S3). The dsRNA molecule in the nsp14–nsp10–RNA complex was stable throughout the simulation with direct RNA phosphate contacts by nsp14 Lys9, Lys61, and the terminal amino group of nsp10 maintained (Fig. 4*B* and *SI Appendix*, Figs. S6 and S11), with small adjustments of atomic positions including a horizontal displacement of the 5′-terminal base of the template strand by His95 (Fig. 6 *F* and *G*). An ionic interaction between Ala1 of nsp10 and RNA backbone phosphate was particularly persistent and observed for 97% of the time during the simulations (3.2-Å distance cutoff), which led to a significant stabilization of this residue in the presence of RNA (Table 3). Lastly, it is also worth noting that an analysis of the internal dynamics of the N7-MTase domain indicates several highly mobile regions, including loops (residues 289 to 300 and 355 to 362) flanking the substrates-binding cleft and a loop (residues 454 to 470) adjacent to the third zinc finger motif of nsp14 distal to the N7-MTase active site (*SI Appendix*, Fig. S12).

**Table 3.** RMSF (in angstroms) of the catalytic residues and RNA-binding residues in the three simulated systems

	nsp14	nsp14–nsp10	nsp14–nsp10–RNA
D90 (nsp14)	0.44 (0.56)	0.40 (0.52)	0.35 (0.39)
E92 (nsp14)	0.61 (1.18)	0.63 (1.16)	0.39 (0.76)
E191 (nsp14)	0.58 (0.76)	0.56 (0.75)	0.38 (0.61)
H268 (nsp14)	1.75 (2.33)	1.66 (2.21)	1.34 (2.05)
D273 (nsp14)	0.58 (0.97)	0.60 (0.92)	0.39 (0.45)
K9 (nsp14)	1.80 (2.59)	1.16 (1.62)	0.55 (0.67)
K61 (nsp14)	2.81 (3.49)	1.60 (2.26)	0.73 (1.25)
K139 (nsp14)	0.95 (1.56)	0.86 (1.52)	0.62 (1.18)
A1 (nsp10)		4.04 (4.12)	0.75 (0.82)

RMSFs of Cα atoms were calculated after aligning trajectories to the initial model with respect to Cα atoms of residues 71 to 289 (core of the ExoN domain). RMSF of all atoms for each residue is presented in parentheses. Catalytic residues of ExoN are underlined.

## Discussion

Our X-ray crystallographic, biochemical, and computational analyses shed light on the substrate preference, structure, and dynamics of the SARS-CoV-2 ExoN–nsp10 complex and further identified important roles of nsp10 in RNA substrate binding. It is particularly notable that the ExoN–nsp10 complex preferentially degrades dsRNA substrates. This is in contrast to the proofreading exonuclease domain of high-fidelity DNA polymerases, whose active site engages the single-stranded DNA 3′ end in partially melted double-stranded substrates (27, 37), and suggests a unique mechanism of proofreading. The extensive ExoN/nsp10 interface buries a total of 2,203 Å^2^ of surfaces from both proteins, spanning both the “fingers” and “palm” regions of ExoN. Folding of the fingers region depends on its interaction with nsp10, which involves several critical residues, including nsp10 Tyr96 (35) (Fig. 3*A* and *SI Appendix*, Fig. S7). On the other hand, an interesting feature for the interaction in the palm region includes the insertion of Phe16 and Phe19 from the first α-helix of nsp10 into a deep hydrophobic pocket of ExoN, which is essential for stable complex formation (35). Notably, this hydrophobic pocket is located on the backside from the ExoN active site, where nsp10 Phe19 side chain makes van der Waals contacts with the main chain of an ExoN α-helix harboring one of the catalytic residues Glu191 (*SI Appendix*, Fig. S7). Thus, targeting said pocket of ExoN by small molecules to block its interaction with nsp10 or potentially to allosterically modulate its catalytic activity could be a possible strategy of inhibition.

MD simulations revealed remarkable flexibility in full-length nsp14 (*SI Appendix*, Figs. S8 and S9 and Movies S1 and S2), which affects solvent accessibility of the SAM/GpppA-binding cleft and may play an important role in the catalytic cycle of N7-MTase (Fig. 6*B*). Similar conformational variation, albeit with a much smaller magnitude, was previously observed between two SARS-CoV nsp14 molecules in the asymmetric unit of a crystal (*SI Appendix*, Fig. S9), and substantial conformational changes between the ExoN and N7-MTase domains were also suggested in solution small angle X-ray scattering studies (21). Although this hinge motion was observed for all three systems (nsp14 alone, nsp14–nsp10, and nsp14–nsp10–RNA) in our simulations, they showed different distributions of the PC1 value (*SI Appendix*, Fig. S8). In addition, conformational sampling in the nsp14-alone system shows several clusters with distinct combinations of PC1 and PC2 values (Fig. 6 *A*, *Left*), suggesting that there may be a long-range interaction between the N-terminal fingers region of ExoN and the C-terminal N7-MTase domain. These observations are consistent with earlier studies showing that single amino acid substitutions R84A and W86A within the ExoN domain completely abolished, while a deletion of the N-terminal 61 residues significantly enhanced, the N7-MTase activity of SARS-CoV nsp14 (38). These mutations in ExoN may have modulated the PC1 motion of nsp14 to affect its N7-MTase activity. Conversely, although we showed, in this study, that the N7-MTase domain is not essential for the ExoN activity of nsp14 in vitro, SARS-CoV nsp14 N7-MTase domain residues Tyr498 and His487 were shown to be required for RdRp/nsp12 binding (21), and recent cryo-EM studies of SARS-CoV-2 replication–transcription complex (RTC) suggest that the nsp14 N7-MTase domain interacts with RdRp and nsp13 RNA helicase to facilitate RTC dimerization and possible proofreading *in trans* (39). Thus, it is likely that the ExoN and N7-MTase domains are functionally dependent on each other in vivo, where proper dynamics may be key to support their respective activities and possible coordination. We hope that our structural and functional studies will help future development of ExoN inhibitors to impede the replication of SARS-CoV-2 and related coronaviruses.

## Methods

### Protein Expression and Purification.

SARS-CoV-2 (GenBank: MN908947.3) nsp14 and its N-terminal ExoN domain, nsp14(1 to 289), were coexpressed with nsp10 in *Escherichia coli* strain BL21(DE3) under the control of T7 promoters. To facilitate purification, a 6xHis tag was added to the N terminus of nsp14 and nsp14(1 to 289) with a human rhinovirus (HRV) 3C protease cleavage site. A methionine residue was added to nsp10 to enable translation. Transformed bacteria were cultured in lysogeny broth medium at 37 °C to the midlog phase, supplemented with 0.5 mM and 50 μM (final concentrations) isopropyl β-D-1-thiogalactopyranoside and zinc chloride, respectively, and further incubated at 18 °C overnight before being pelleted by centrifugation. Collected bacteria were disrupted by the addition of hen egg white lysozyme and sonication in 20 mM Tris⋅HCl, pH 7.4, 0.5 M NaCl, 5 mM β-mercaptoethanol, and 5 mM imidazole. The lysate was cleared by centrifugation at 63,000 × *g* for 1 h at 4 °C, after which the protein complex in the supernatant was captured by nickel-affinity chromatography and eluted by a linear gradient of imidazole. Eluted proteins were digested with HRV 3C protease overnight at 4 °C, concentrated by ultrafiltration, and passed through a Superdex75 size exclusion column operating with the same buffer as above except not containing imidazole. The nsp14–nsp10 complexes eluted as a heterodimer were concentrated by ultrafiltration and frozen in small-volume aliquots in liquid nitrogen for storage at −80 °C. The ExoN mutant derivatives were generated by site-directed mutagenesis and purified using the same procedure. Full-length SARS-CoV-2 nsp12 was expressed with an N-terminal 10xHis-small ubiquitin-like modifier (SUMO) tag in *E. coli* strain BL21(DE3) and purified as described above, except that the SUMO tag was not cleaved. SARS-CoV-2 nsp8 with an N-terminal 10xHis-SUMO tag was coexpressed with nsp7 with an additional methionine at the N terminus, and the complex was purified as above except that the SUMO tag was cleaved by Ulp1 protease prior to the size exclusion step. The protein concentrations were determined based on ultraviolet absorbance at 280 nm measured on a Nanodrop8000 spectrophotometer and theoretical extinction coefficients calculated from the protein amino acid sequences. Concentration of the nsp7–nsp8 complex was calculated based on an assumed 1:2 stoichiometry.

### Crystallization and Structure Determination.

Purified nsp14(1 to 289, E191Q)–nsp10 complex (*SI Appendix*, Fig. S13) at 17 mg⋅mL^−1^ was crystallized using the hanging drop vapor diffusion method, by mixing the protein solution with an equal volume of reservoir solution including either 0.2 M di-ammonium tartrate, pH 7.0, 20% polyethylene glycol (PEG) 3,350 (condition 1), or 0.1 M MgCl_2_, 0.1 M Tris⋅HCl pH 8.5, 20% PEG 4,000 (condition 2). Both conditions produced thin needle crystals. The crystals were cryoprotected with ethylene glycol and flash cooled by plunging in liquid nitrogen. X-ray diffraction data were collected at the Northeastern Collaborative Access Team beamlines of the Advanced Photon Source and processed using XDS (40). The structure of the SARS-CoV-2 ExoN–nsp10 complex was determined by molecular replacement phasing by PHASER (41), using the crystal structure of SARS-CoV nsp14–nsp10 complex (PDB ID: 5C8T) (26) as the search model. Iterative model building and refinement were performed using COOT (42) and PHENIX (43), respectively. A summary of data collection and model refinement statistics is shown in Table 2. Structure images were generated using PyMOL (https://pymol.org/).

### Exonuclease Activity Assays.

The 5′-fluorescein–labeled oligonucleotides (Table 1) at 750 nM, in the presence or absence of equimolar complementary unlabeled strands, were incubated with 50 nM (10.5 nM in the experiment shown in Fig. 2*A*) nsp14–nsp10 or nsp14(1 to 289)–nsp10 complex in 42 mM Tris⋅HCl, pH 8.0, 0.94 mM MgCl_2_, 0.94 mM dithiothreitol (DTT), and 0.009% Tween-20. After incubation at 37 °C for 10 min, the reactions were stopped by the addition of formamide to 67% and heating to 95 °C for 10 min. The reaction products were separated on a 15% Tris-borate-EDTA (TBE)-Urea polyacrylamide gel, which was scanned on a Typhoon FLA 9500 imager.

### Sofosbuvir Rescue.

The 5′-fluorescein-labeled oligonucleotide, LS2U, was prepared to 750 nM with an equimolar amount of complementary strand LS15A-RNA (Table 1) and was then added to a reaction buffer containing 20 mM Hepes, pH 7.5, 5 mM MgCl_2_, 10 mM DTT, and 0.01% Tween 20. Addition of 1 μM nsp12, 2 μM nsp7–nsp8 complex, and an rNTP mix at 10× the concentration relative to the unpaired template bases—15 μM CTP, 30 μM sofosbuvir 5′-triphosphate (Sierra Bioresearch), 60 μM ATP, and 45 μM GTP—was followed by incubation at 37 °C for 20 min, to yield stalled RNA products. Then, 30 μM UTP and 10 nM nsp14–nsp10 or 70 nM nsp14(1 to 289)–nsp10 complex were added and allowed to further incubate at 37 °C for 30 min. In control reactions, either UTP or enzyme was omitted. The reactions were stopped by the addition of formamide to 90% and heating to 95 °C for 10 min. The reaction products were separated on a 15% TBE-Urea polyacrylamide gel, which was scanned on a Typhoon FLA 9500 imager.

### Degradation of Remdesivir-Containing RNA.

The 5′-fluorescein-labeled oligonucleotide, LS2U, was prepared to 750 nM with an equimolar amount of complementary strand LS15A-RNA (Table 1) and was then added to a reaction buffer containing 20 mM Hepes, pH 7.5, 5 mM MgCl_2_, 10 mM DTT, and 0.01% Tween 20. Addition of 1 μM nsp12, 2 μM nsp7–nsp8 complex, and an rNTP mix at 10× the concentration relative to the unpaired template bases—15 μM CTP, 30 μM UTP, 60 μM GS 441524 triphosphate (Biosynth Carbosynth), and 45 μM GTP—was followed by incubation at 37 °C for 20 min, to yield fully or partially extended RNA products. For a control reaction, ATP was substituted for GS 441524 triphosphate. The reactions were treated at 95 °C for 5 min to inactivate nsp12. Then, nsp14–nsp10 or nsp14(1 to 289)–nsp10 complex was added at 200 nM or 75 nM, and degradation was allowed to proceed for 10 min at 37 °C. The reactions were stopped by the addition of formamide to 90% and heating to 95 °C for 10 min. The reaction products were separated on a 15% TBE-Urea polyacrylamide gel, which was scanned on a Typhoon FLA 9500 imager. To confirm the incorporation of remdesivir during primer extension by RdRp, a 50-μL aliquot of each extension product was analyzed by an Orbitrap Fusion mass spectrometer (Thermo Scientific) connected to an Acquity i-Class ultra performance liquid chromatography (UPLC, Waters). Mass resolution was set to 60,000. Data were processed using Novatia ProMass HR software, which uses the PPL ReSpect deisotoping algorithm to determine monoisotopic masses of the multiply charged oligonucleotides. Gradient UPLC conditions were as follows: column: 2.1 × 50 mm Acquity ethylene bridged hybrid C18 130 Å, 1.7 μm (Waters); temperature: 60 °C; flow rate: 0.4 mL/min, 5%B at 0 min, 10%B at 1 min, 20% B at 15 min, 40%B at 20 min, 65%B at 21 min; A: 1% HFIPA (hexafluoroisopropanol)/0.1% DIEA (diisopropylethylamine) in water; B: 65/35 acetonitrile/water with 0.075% HFIPA/0.0375% DIEA.

### MD Simulations.

A homology model of full-length SARS-CoV-2 nsp14 was generated for sequence of YP_009725309.1 and taking SARS-CoV nsp14 crystal structure (PDB ID: 5NFY) (21) as a template in Schrödinger Prime module (44). The nsp14 ExoN domain of the homology model was then replaced with the crystal structure of SARS-CoV-2 nsp14 ExoN in complex with nsp10 obtained in this study. E191Q mutation in the crystal structure was reverted computationally to the wild type. For an nsp14–nsp10–RNA model, RNA was modeled based on Lassa NP-RNA complex (PDB ID: 4FVU) (30), and the second Mg ion at the active site was modeled based on a Mn^2+^ ion found in Lassa NP-RNA complex (PDB ID: 4GV9) (31). Three systems were prepared from this model: 1) full-length nsp14 alone, 2) full-length nsp14–nsp10 complex, and 3) full-length nsp14–nsp10–RNA complex. Protonation states of titratable amino acids were determined using PropKa analysis (45). Each of these systems was explicitly solvated in TIP3P water box, and ions were added to achieve 0.2 M salt concentration. Amber ff14SB (46) and RNA.OL3 force fields are used for protein and RNA, respectively. For zinc ions and zinc-coordinating residues, Cationic Dummy Atom parameters were used (47). Conventional MD simulations (cMD) were performed with the NAMD2.14 program (48), while GAMD were performed with the Amber20 program (49). First, each system was minimized in four consecutive steps by gradually decreasing restraints. Subsequently, each system was heated from 0 K to 310 K slowly, and then equilibrated for about 1 ns by gradually decreasing restraints in three consecutive steps. For cMD, three independent copies (2× 1 µs and 1× 0.6 µs) of simulation were run for each system. For GAMD, three independent copies of 0.2 μs of simulation were run for each system using the dual boost method following a 20-ns MD run to calculate parameters for GAMD production runs. All cMD and GAMD simulations were performed at 310 K and 1 atm and with a 2-fs time step. For each system, 32,000 data points with 0.1-ns intervals were collected from simulations and analyzed. Stability of MD simulations is shown with rmsd plots of the nsp14 ExoN domain (*SI Appendix*, Fig. S14). MDTraj (50) was used for some of the MD trajectory analysis.

## Supplementary Material

Supplementary File

Supplementary File

Supplementary File

Supplementary File

## Data Availability

The atomic coordinates and structure factors for the SARS-CoV-2 ExoN–nsp10 complex structures have been deposited in the Research Collaboratory for Structural Bioinformatics Protein Data Bank, with the accession codes 7MC5 and 7MC6. In accordance with COVID-19 community sharing principles (51), the MD simulation data, including all input and resulting trajectory files, are available on the NSF MolSSI COVID-19 Molecular Structure and Therapeutics Hub at https://covid.molssi.org.

## References

[1] H. S. Hillen , Structure of replicating SARS-CoV-2 polymerase. Nature 584, 154–156 (2020). 32438371 10.1038/s41586-020-2368-8

[2] C. C. Posthuma, A. J. W. Te Velthuis, E. J. Snijder, Nidovirus RNA polymerases: Complex enzymes handling exceptional RNA genomes. Virus Res. 234, 58–73 (2017). 28174054 10.1016/j.virusres.2017.01.023 PMC7114556

[3] I. Sola, F. Almazán, S. Zúñiga, L. Enjuanes, Continuous and discontinuous RNA synthesis in coronaviruses. Annu. Rev. Virol. 2, 265–288 (2015). 26958916 10.1146/annurev-virology-100114-055218 PMC6025776

[4] J. W. Drake, J. J. Holland, Mutation rates among RNA viruses. Proc. Natl. Acad. Sci. U.S.A. 96, 13910–13913 (1999). 10570172 10.1073/pnas.96.24.13910 PMC24164

[5] G. M. Jenkins, A. Rambaut, O. G. Pybus, E. C. Holmes, Rates of molecular evolution in RNA viruses: A quantitative phylogenetic analysis. J. Mol. Evol. 54, 156–165 (2002). 11821909 10.1007/s00239-001-0064-3

[6] R. Sanjuán, M. R. Nebot, N. Chirico, L. M. Mansky, R. Belshaw, Viral mutation rates. J. Virol. 84, 9733–9748 (2010). 20660197 10.1128/JVI.00694-10 PMC2937809

[7] M. R. Denison, R. L. Graham, E. F. Donaldson, L. D. Eckerle, R. S. Baric, Coronaviruses: An RNA proofreading machine regulates replication fidelity and diversity. RNA Biol. 8, 270–279 (2011). 21593585 10.4161/rna.8.2.15013 PMC3127101

[8] A. E. Gorbalenya, L. Enjuanes, J. Ziebuhr, E. J. Snijder, Nidovirales: Evolving the largest RNA virus genome. Virus Res. 117, 17–37 (2006). 16503362 10.1016/j.virusres.2006.01.017 PMC7114179

[9] A. Shannon , Rapid incorporation of Favipiravir by the fast and permissive viral RNA polymerase complex results in SARS-CoV-2 lethal mutagenesis. Nat. Commun. 11, 4682 (2020). 32943628 10.1038/s41467-020-18463-z PMC7499305

[10] E. Minskaia , Discovery of an RNA virus 3′->5′ exoribonuclease that is critically involved in coronavirus RNA synthesis. Proc. Natl. Acad. Sci. U.S.A. 103, 5108–5113 (2006). 16549795 10.1073/pnas.0508200103 PMC1458802

[11] F. Robson , Coronavirus RNA proofreading: Molecular basis and therapeutic targeting. Mol. Cell 79, 710–727 (2020). 32853546 10.1016/j.molcel.2020.07.027 PMC7402271

[12] E. C. Smith, M. R. Denison, Coronaviruses as DNA wannabes: A new model for the regulation of RNA virus replication fidelity. PLoS Pathog. 9, e1003760 (2013). 24348241 10.1371/journal.ppat.1003760 PMC3857799

[13] D. Eskier, A. Suner, Y. Oktay, G. Karakülah, Mutations of SARS-CoV-2 nsp14 exhibit strong association with increased genome-wide mutation load. PeerJ 8, e10181 (2020). 33083157 10.7717/peerj.10181 PMC7560320

[14] K. Takada, M. Takahashi Ueda, T. Watanabe, S. Nakagawa, Genomic diversity of SARS-CoV-2 can be accelerated by a mutation in the nsp14 gene. bioRxiv [Preprint] (2020). 10.1101/2020.12.23.424231 (Accessed 6 February 2022). PMC9933857 36811085

[15] L. D. Eckerle , Infidelity of SARS-CoV Nsp14-exonuclease mutant virus replication is revealed by complete genome sequencing. PLoS Pathog. 6, e1000896 (2010). 20463816 10.1371/journal.ppat.1000896 PMC2865531

[16] L. D. Eckerle, X. Lu, S. M. Sperry, L. Choi, M. R. Denison, High fidelity of murine hepatitis virus replication is decreased in nsp14 exoribonuclease mutants. J. Virol. 81, 12135–12144 (2007). 17804504 10.1128/JVI.01296-07 PMC2169014

[17] R. L. Graham , A live, impaired-fidelity coronavirus vaccine protects in an aged, immunocompromised mouse model of lethal disease. Nat. Med. 18, 1820–1826 (2012). 23142821 10.1038/nm.2972 PMC3518599

[18] N. S. Ogando , The enzymatic activity of the nsp14 exoribonuclease is critical for replication of MERS-CoV and SARS-CoV-2. J. Virol. 94, e01246-20 (2020). 10.1128/JVI.01246-20 PMC7654266 32938769

[19] J. Gribble , The coronavirus proofreading exoribonuclease mediates extensive viral recombination. PLoS Pathog. 17, e1009226 (2021). 33465137 10.1371/journal.ppat.1009226 PMC7846108

[20] J. B. Case , Murine hepatitis virus nsp14 exoribonuclease activity is required for resistance to innate immunity. J. Virol. 92, e01531-17 (2017). 29046453 10.1128/JVI.01531-17 PMC5730787

[21] F. Ferron , Structural and molecular basis of mismatch correction and ribavirin excision from coronavirus RNA. Proc. Natl. Acad. Sci. U.S.A. 115, E162–E171 (2018). 29279395 10.1073/pnas.1718806115 PMC5777078

[22] M. L. Agostini , Coronavirus susceptibility to the antiviral Remdesivir (GS-5734) is mediated by the viral polymerase and the proofreading exoribonuclease. MBio 9, e00221-18 (2018). 29511076 10.1128/mBio.00221-18 PMC5844999

[23] E. C. Smith, H. Blanc, M. C. Surdel, M. Vignuzzi, M. R. Denison, Coronaviruses lacking exoribonuclease activity are susceptible to lethal mutagenesis: Evidence for proofreading and potential therapeutics. PLoS Pathog. 9, e1003565 (2013). 23966862 10.1371/journal.ppat.1003565 PMC3744431

[24] C. Q. Sacramento , In vitro antiviral activity of the anti-HCV drugs daclatasvir and sofosbuvir against SARS-CoV-2, the aetiological agent of COVID-19. J. Antimicrob. Chemother. 76, 1874–1885 (2021). 33880524 10.1093/jac/dkab072 PMC8083231

[25] S. Jockusch , Sofosbuvir terminated RNA is more resistant to SARS-CoV-2 proofreader than RNA terminated by Remdesivir. Sci. Rep. 10, 16577 (2020). 33024223 10.1038/s41598-020-73641-9 PMC7538426

[26] Y. Ma , Structural basis and functional analysis of the SARS coronavirus nsp14-nsp10 complex. Proc. Natl. Acad. Sci. U.S.A. 112, 9436–9441 (2015). 26159422 10.1073/pnas.1508686112 PMC4522806

[27] L. S. Beese, T. A. Steitz, Structural basis for the 3′-5′ exonuclease activity of *Escherichia coli* DNA polymerase I: A two metal ion mechanism. EMBO J. 10, 25–33 (1991). 1989886 10.1002/j.1460-2075.1991.tb07917.x PMC452607

[28] P. Chen , Biochemical characterization of exoribonuclease encoded by SARS coronavirus. J. Biochem. Mol. Biol. 40, 649–655 (2007). 17927896 10.5483/bmbrep.2007.40.5.649

[29] X. Qi , Cap binding and immune evasion revealed by Lassa nucleoprotein structure. Nature 468, 779–783 (2010). 21085117 10.1038/nature09605 PMC3057469

[30] K. M. Hastie, L. B. King, M. A. Zandonatti, E. O. Saphire, Structural basis for the dsRNA specificity of the Lassa virus NP exonuclease. PLoS One 7, e44211 (2012). 22937163 10.1371/journal.pone.0044211 PMC3429428

[31] X. Jiang , Structures of arenaviral nucleoproteins with triphosphate dsRNA reveal a unique mechanism of immune suppression. J. Biol. Chem. 288, 16949–16959 (2013). 23615902 10.1074/jbc.M112.420521 PMC3675627

[32] M. Bouvet , RNA 3′-end mismatch excision by the severe acute respiratory syndrome coronavirus nonstructural protein nsp10/nsp14 exoribonuclease complex. Proc. Natl. Acad. Sci. U.S.A. 109, 9372–9377 (2012). 22635272 10.1073/pnas.1201130109 PMC3386072

[33] C. Liu , Structural basis of mismatch recognition by a SARS-CoV-2 proofreading enzyme. Science 373, 1142–1146 (2021). 34315827 10.1126/science.abi9310 PMC9836006

[34] M. Bouvet , Coronavirus Nsp10, a critical co-factor for activation of multiple replicative enzymes. J. Biol. Chem. 289, 25783–25796 (2014). 25074927 10.1074/jbc.M114.577353 PMC4162180

[35] M. Saramago , New targets for drug design: Importance of nsp14/nsp10 complex formation for the 3′-5′ exoribonucleolytic activity on SARS-CoV-2. *FEBS J.* **288**, 5130–5147 (2021). 10.1111/febs.15815 PMC8237063 33705595

[36] H. T. Baddock , Characterisation of the SARS-CoV-2 ExoN (nsp14^ExoN^-nsp10) complex: Implications for its role in viral genome stability and inhibitor identification. *Nucleic Acids Res.*, 10.1093/nar/gkab1303 (2022). PMC8860572 35037045

[37] L. S. Beese, V. Derbyshire, T. A. Steitz, Structure of DNA polymerase I Klenow fragment bound to duplex DNA. Science 260, 352–355 (1993). 8469987 10.1126/science.8469987

[38] Y. Chen , Structure-function analysis of severe acute respiratory syndrome coronavirus RNA cap guanine-N7-methyltransferase. J. Virol. 87, 6296–6305 (2013). 23536667 10.1128/JVI.00061-13 PMC3648086

[39] L. Yan , Coupling of N7-methyltransferase and 3′-5′ exoribonuclease with SARS-CoV-2 polymerase reveals mechanisms for capping and proofreading. Cell 184, 3474–3485.e11 (2021). 34143953 10.1016/j.cell.2021.05.033 PMC8142856

[40] W. Kabsch, XDS. Acta Crystallogr. D Biol. Crystallogr. 66, 125–132 (2010). 20124692 10.1107/S0907444909047337 PMC2815665

[41] A. J. McCoy , Phaser crystallographic software. J. Appl. Cryst. 40, 658–674 (2007). 19461840 10.1107/S0021889807021206 PMC2483472

[42] P. Emsley, B. Lohkamp, W. G. Scott, K. Cowtan, Features and development of Coot. Acta Crystallogr. D Biol. Crystallogr. 66, 486–501 (2010). 20383002 10.1107/S0907444910007493 PMC2852313

[43] D. Liebschner , Macromolecular structure determination using X-rays, neutrons and electrons: Recent developments in Phenix. Acta Crystallogr. D Struct. Biol. 75, 861–877 (2019). 31588918 10.1107/S2059798319011471 PMC6778852

[44] Schrödinger, Schrödinger (Release 2021-1: Prime, Schrödinger, LLC, New York, NY, 2021).

[45] T. J. Dolinsky, J. E. Nielsen, J. A. McCammon, N. A. Baker, PDB2PQR: An automated pipeline for the setup of Poisson-Boltzmann electrostatics calculations. Nucleic Acids Res. 32, W665–W667 (2004). 15215472 10.1093/nar/gkh381 PMC441519

[46] J. A. Maier , ff14SB: Improving the accuracy of protein side chain and backbone parameters from ff99SB. J. Chem. Theory Comput. 11, 3696–3713 (2015). 26574453 10.1021/acs.jctc.5b00255 PMC4821407

[47] Y. P. Pang, Novel zinc protein molecular dynamics simulations: Steps toward antiangiogenesis for cancer treatment. J. Mol. Model. 5, 196–202 (1999).

[48] J. C. Phillips , Scalable molecular dynamics on CPU and GPU architectures with NAMD. J. Chem. Phys. 153, 044130 (2020). 32752662 10.1063/5.0014475 PMC7395834

[49] D. A. Case , AMBER 2020 (University of California, San Francisco, 2020).

[50] R. T. McGibbon , MDTraj: A modern open library for the analysis of molecular dynamics trajectories. Biophys. J. 109, 1528–1532 (2015). 26488642 10.1016/j.bpj.2015.08.015 PMC4623899

[51] R. E. Amaro, A. J. Mulholland, A community letter regarding sharing biomolecular simulation data for COVID-19. J. Chem. Inf. Model. 60, 2653–2656 (2020). 32255648 10.1021/acs.jcim.0c00319

[52] N. A. Baker, D. Sept, S. Joseph, M. J. Holst, J. A. McCammon, Electrostatics of nanosystems: Application to microtubules and the ribosome. Proc. Natl. Acad. Sci. U.S.A. 98, 10037–10041 (2001). 11517324 10.1073/pnas.181342398 PMC56910

