# Supplementary material for: Structure and dynamics of SARS-CoV-2 proofreading exoribonuclease ExoN
Source: Proc Natl Acad Sci U S A. 2022 Feb 14;119(9):e2106379119. doi: 10.1073/pnas.2106379119 (PMC8892293; doi:10.1073/pnas.2106379119)
Supplement: Supplementary File [file pnas.2106379119.sapp.pdf]

PNAS
www.pnas.org

**Supplementary Information for**

Structure and dynamics of SARS-CoV-2 proofreading exoribonuclease ExoN

Nicholas H. Moeller[1,2,3†], Ke Shi[1,2,3†], Özlem Demir[4†], Christopher Belica[1,2,3], Surajit Banerjee[5], Lulu Yin[1,2,3], Cameron Durfee[1,2,3], Rommie E. Amaro[4], Hideki Aihara[1,2,3*]

[1]Department of Biochemistry, Molecular Biology and Biophysics, University of Minnesota, Minneapolis, Minnesota, 55455, USA
[2]Institute for Molecular Virology, University of Minnesota, Minneapolis, Minnesota, 55455, USA
[3]Masonic Cancer Center, University of Minnesota, Minneapolis, Minnesota, 55455, USA
[4]Department of Chemistry and Biochemistry, University of California, San Diego, La Jolla, CA 92093, USA
[5]Northeastern Collaborative Access Team, Cornell University, Advanced Photon Source, Lemont, IL 60439, USA
[†] Co-first authors
*Correspondence: aihar001@umn.edu

**This PDF file includes:**

Figures S1 to S14
Legends for Supplementary animations 1 to 3
SI References

**Other supplementary materials for this manuscript include the following:**

Supplementary animations 1 to 3

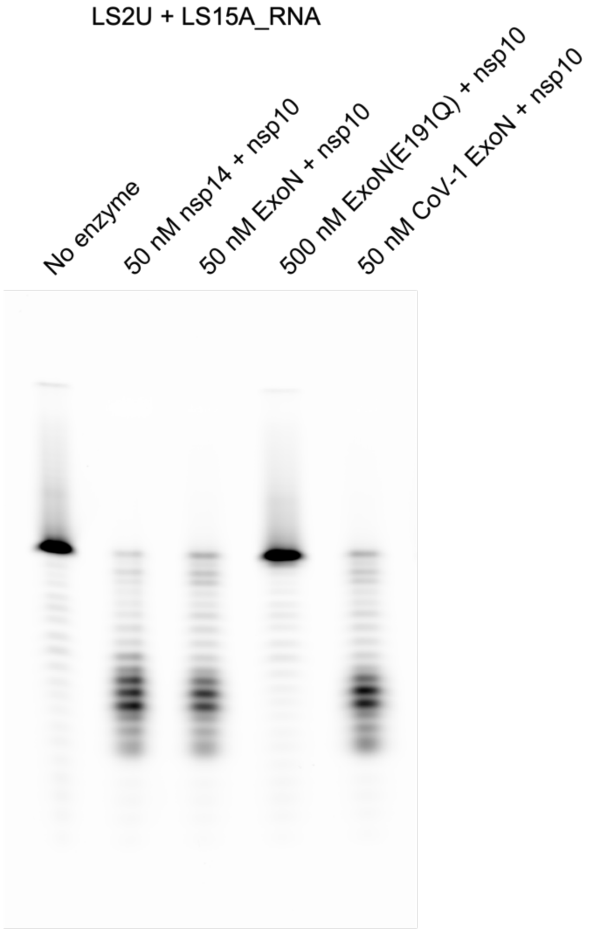


**Fig. S1. Comparison of exoribonuclease activities**

Exoribonuclease activities of SARS-CoV-2 nsp14-nsp10, ExoN-nsp10, and SARS-CoV ExoN-nsp10 complexes on a double-stranded RNA substrate. The inactive E191Q mutant enzyme was tested at a 10 times higher protein concentration.

**A**

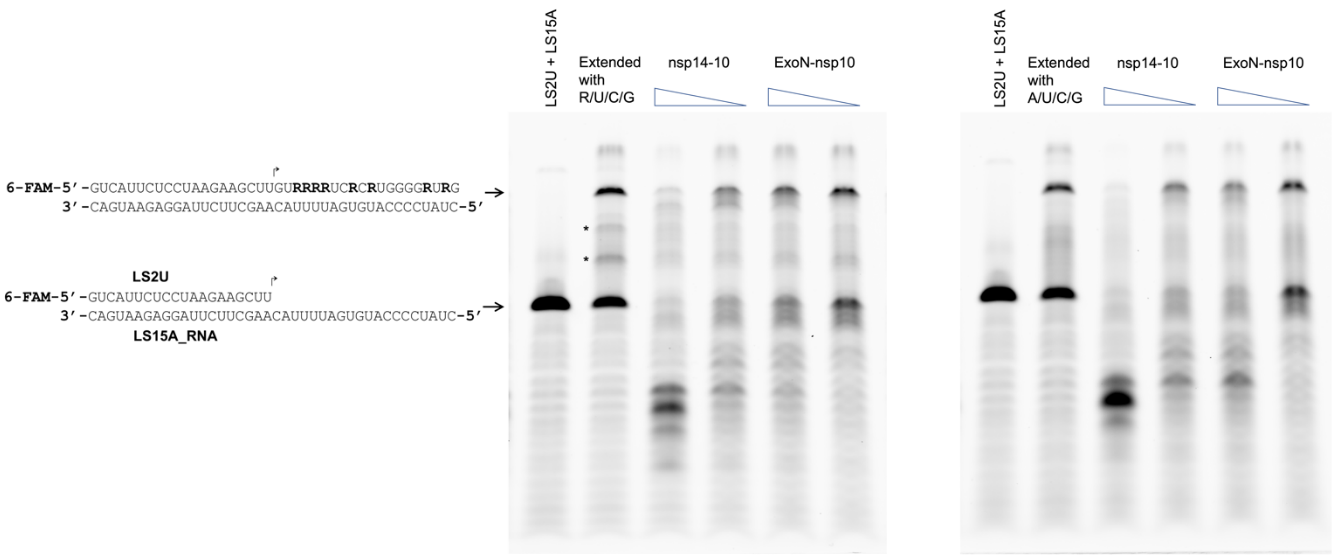


**Fig. S2. Degradation of remdesivir-containing RNA by nsp14/ExoN**

**A**, The LS2U primer extended by RdRp with remdesivir triphosphate in place of ATP (left) or with natural NTPs (right) were incubated with 200 or 75 nM nsp14-nsp10 or ExoN-nsp10 complex. Asterisks mark abortive extension products.

**B (below)**, Liquid chromatography mass spectrometry (LC-MS) analysis of the LS2U primer extended by SARS-CoV-2 RdRp in the presence of remdesivir triphosphate in place of ATP. Deconvoluted mass spectra for indicated retention times (RT) are shown below the total ion chromatogram and LC/UV chromatogram at the top. The observed monoisotopic mass of the major extension product (13551.883 Da) closely matches the theoretical value for a full remdesivir incorporation (13551.840 Da).

**C (below)**, LC-MS analysis of the LS2U primer extended by SARS-CoV-2 RdRp in the presence of natural NTPs. The observed monoisotopic mass of the major extension product (13359.882 Da) closely matches the theoretical value (13359.840 Da).

Fig. S2B

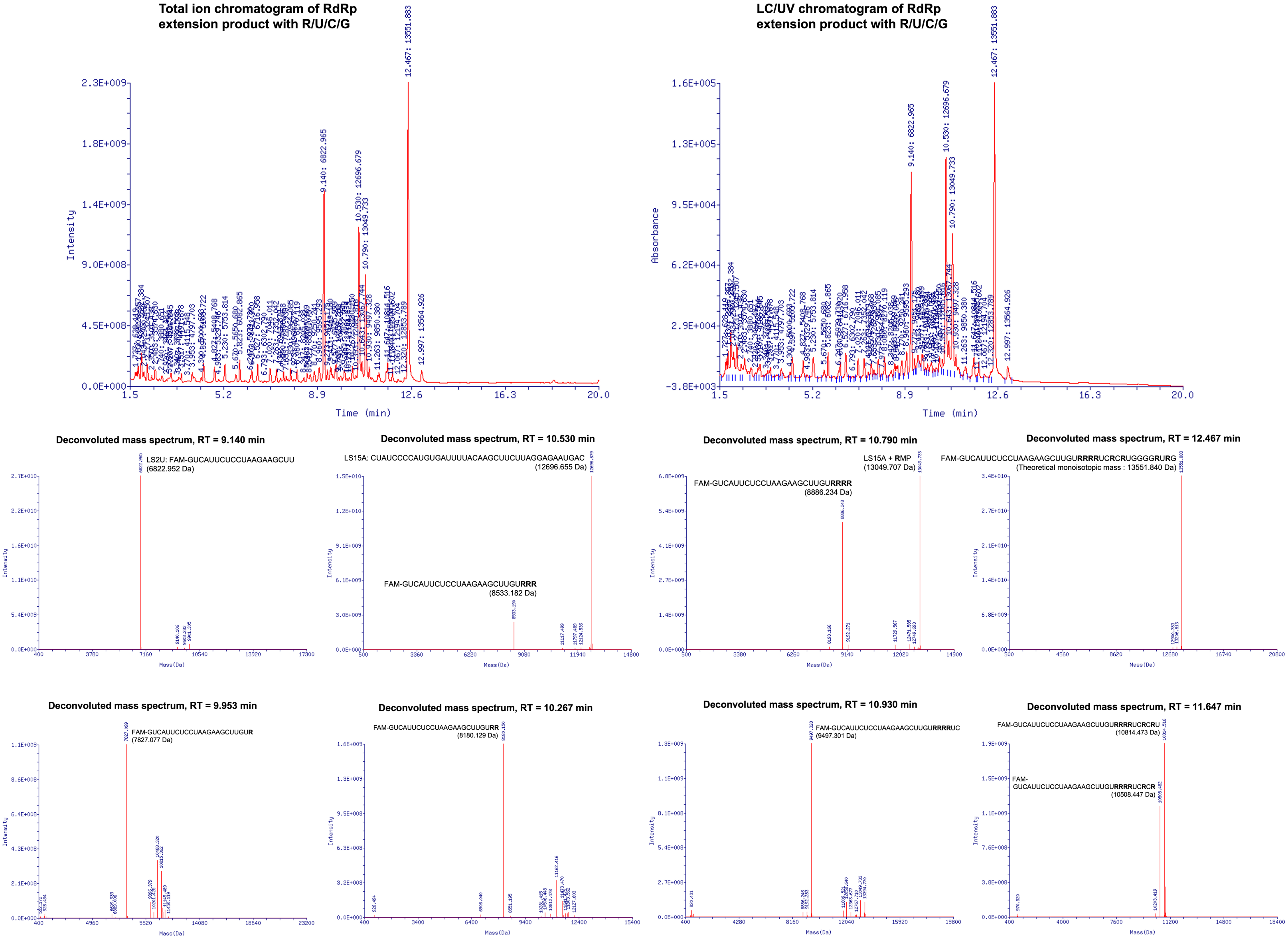

Total ion chromatogram of RdRp
extension product with R/U/C/G
LC/UV chromatogram of RdRp
extension product with R/U/C/G
12.467: 13551.883
9.140: 6822.965
10.530: 12696.679
10.790: 13049.733
Intensity
Absorbance
Time (min)
Deconvoluted mass spectrum, RT = 9.140 min
LS2U: FAM-GUCAUUCUCCUAAGAAGCUU
(6822.952 Da)
Deconvoluted mass spectrum, RT = 10.530 min
LS15A: CUAUCCCCAUGUGAUUUUACAAGCUUCUUAGGAGAAUGAC
(12696.655 Da)
FAM-GUCAUUCUCCUAAGAAGCUUGURRR
(8533.182 Da)
Deconvoluted mass spectrum, RT = 10.790 min
LS15A + RMP
(13049.707 Da)
FAM-GUCAUUCUCCUAAGAAGCUUGURRRR
(8886.234 Da)
Deconvoluted mass spectrum, RT = 12.467 min
FAM-GUCAUUCUCCUAAGAAGCUUGURRRRUCRCRUGGGGRURG
(Theoretical monoisotopic mass : 13551.840 Da)
Deconvoluted mass spectrum, RT = 9.953 min
FAM-GUCAUUCUCCUAAGAAGCUUGUR
(7827.077 Da)
Deconvoluted mass spectrum, RT = 10.267 min
FAM-GUCAUUCUCCUAAGAAGCUUGURR
(8180.129 Da)
Deconvoluted mass spectrum, RT = 10.930 min
FAM-GUCAUUCUCCUAAGAAGCUUGURRRRUC
(9497.301 Da)
Deconvoluted mass spectrum, RT = 11.647 min
FAM-GUCAUUCUCCUAAGAAGCUUGURRRRUCRCRU
(10814.473 Da)
FAM-
GUCAUUCUCCUAAGAAGCUUGURRRRUCRCR
(10508.447 Da)
Mass(Da)

Fig. S2C

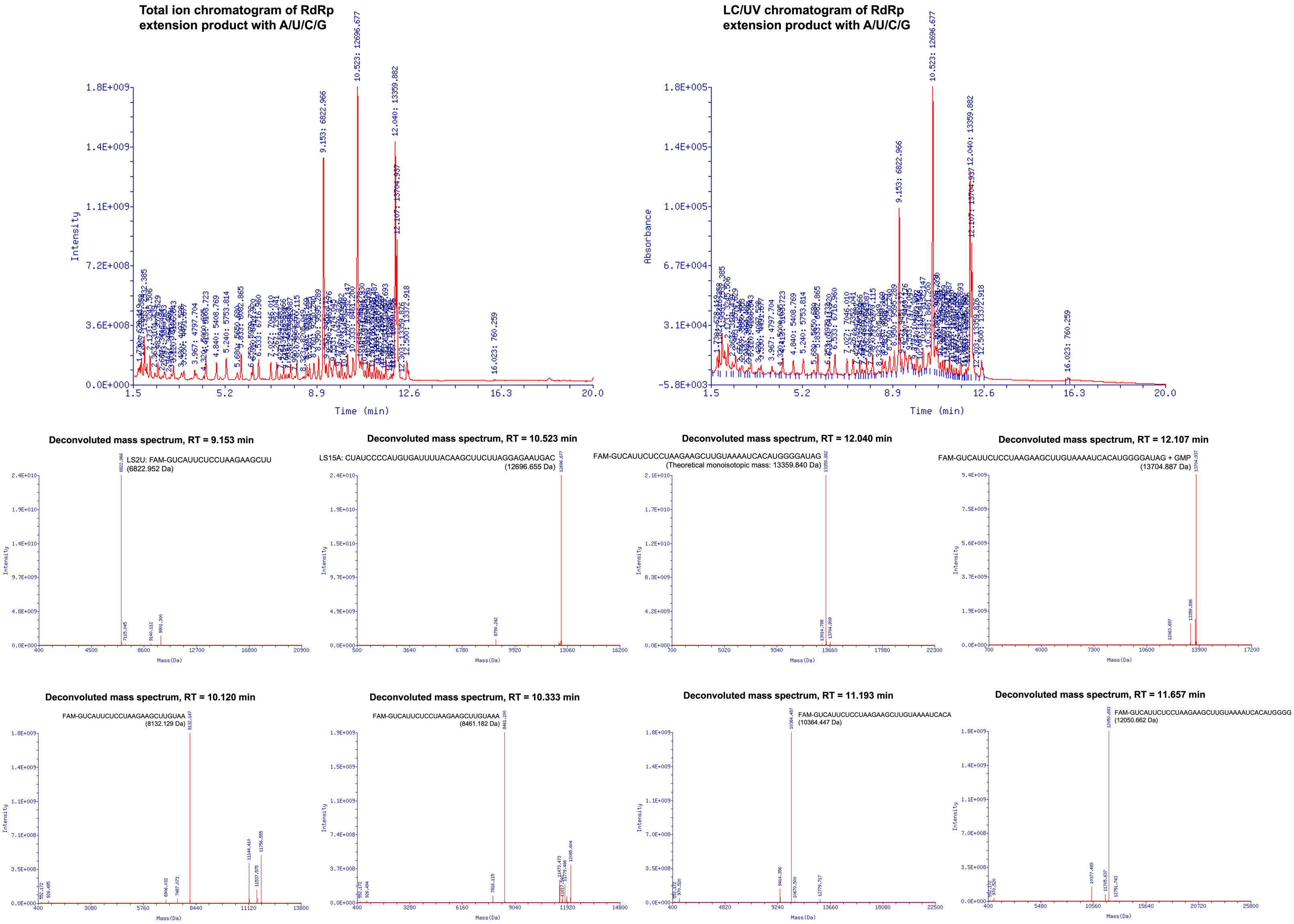
Total ion chromatogram of RdRp extension product with A/U/C/G
LC/UV chromatogram of RdRp extension product with A/U/C/G
Intensity
Absorbance
Time (min)
9.153: 6822.966
10.523: 12696.677
12.040: 13359.882
12.107: 13704.937
16.023: 760.259
Deconvoluted mass spectrum, RT = 9.153 min
LS2U: FAM-GUCAUUCUCCUAAGAAGCUU
(6822.952 Da)
Deconvoluted mass spectrum, RT = 10.523 min
LS15A: CUAUCCCCAUGUGAUUUUACAAGCUUCUUAGGAGAAUGAC
(12696.655 Da)
Deconvoluted mass spectrum, RT = 12.040 min
FAM-GUCAUUCUCCUAAGAAGCUUGUAAAAUCACAUGGGGAUAG
(Theoretical monoisotopic mass: 13359.840 Da)
Deconvoluted mass spectrum, RT = 12.107 min
FAM-GUCAUUCUCCUAAGAAGCUUGUAAAAUCACAUGGGGAUAG + GMP
(13704.887 Da)
Deconvoluted mass spectrum, RT = 10.120 min
FAM-GUCAUUCUCCUAAGAAGCUUGUAA
(8132.129 Da)
Deconvoluted mass spectrum, RT = 10.333 min
FAM-GUCAUUCUCCUAAGAAGCUUGUAAA
(8461.182 Da)
Deconvoluted mass spectrum, RT = 11.193 min
FAM-GUCAUUCUCCUAAGAAGCUUGUAAAAUCACA
(10364.447 Da)
Deconvoluted mass spectrum, RT = 11.657 min
FAM-GUCAUUCUCCUAAGAAGCUUGUAAAAUCACAUGGGG
(12050.662 Da)
Mass(Da)

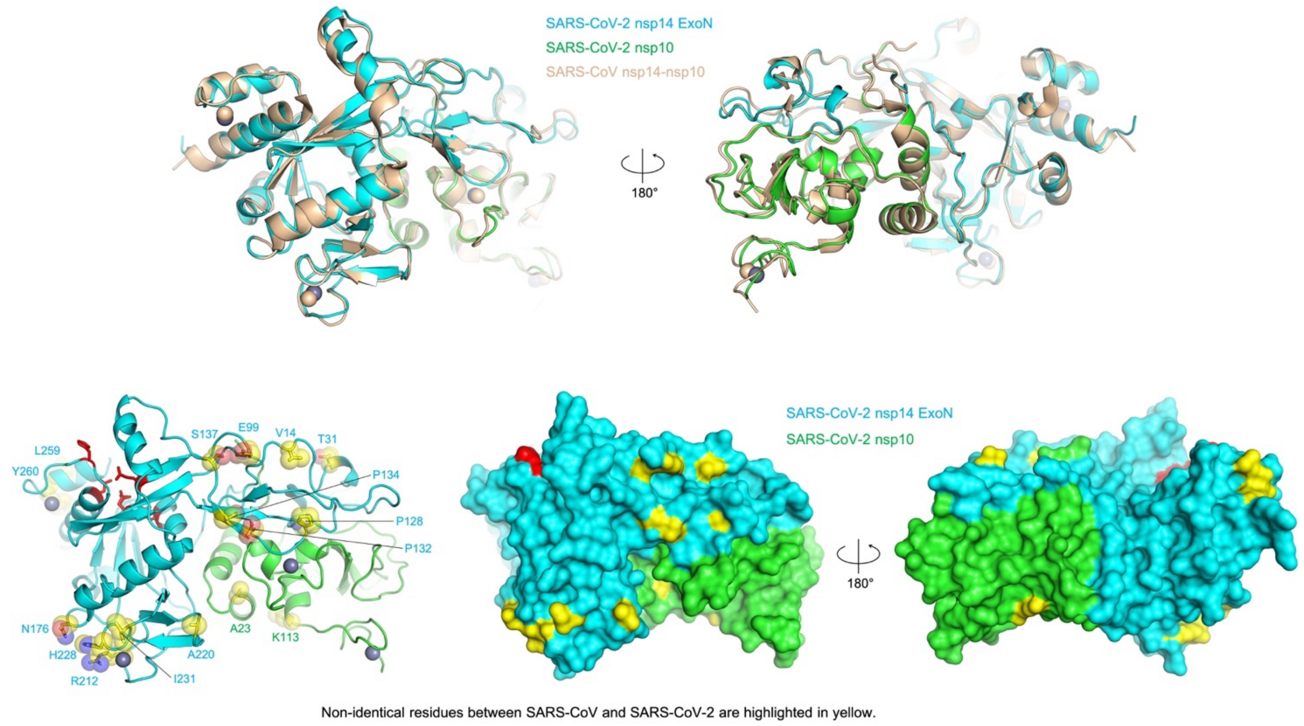


**Fig. S3. SARS-CoV vs. SARS-CoV-2 ExoN-nsp10 structure comparison**

**Top**, A superposition between SARS-CoV (PDB ID: 5C8T) (1) and SARS-CoV-2 (this study) ExoN-nsp10 structures. **Bottom**, Difference in the amino acid sequence between SARS-CoV and SARS-CoV-2 mapped on the ExoN-nsp10 structure and highlighted in yellow. The active site residues are shown in red.

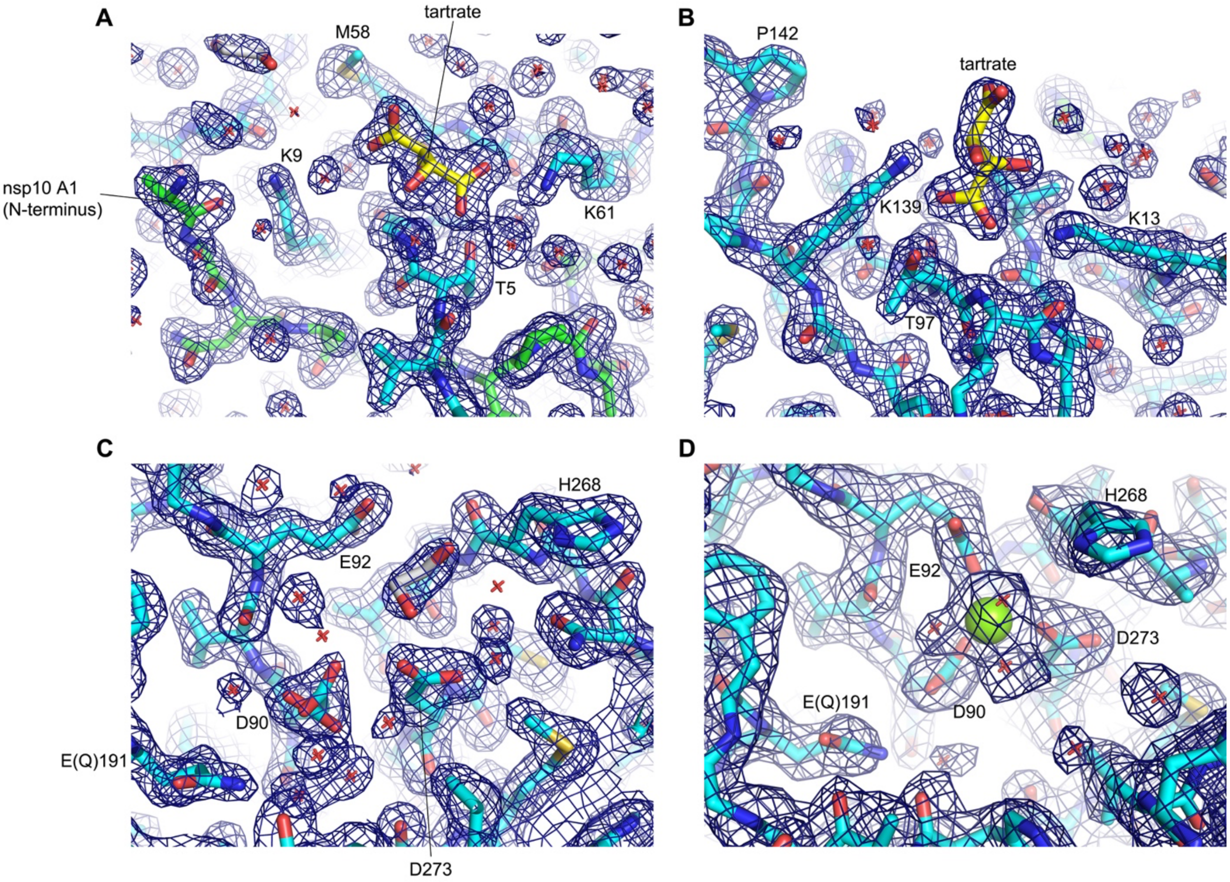


**Fig. S4. Electron density maps**

The 2mFo-DFc map contoured at 1.0 σ is shown for the higher resolution (1.64 Å, PDB ID: 7MC5) tartrate-bound structure in **A-C**, and for the lower resolution (2.10 Å, 7MC6) $Mg^{2+}$-bound structure in **D**.

**A**, Region including Lys9 and Lys61 of nsp14/ExoN and the N-terminus of nsp10 (The crystallized protein has additional methionine residue on the N-terminus, which is likely to be disordered) with a bound tartrate molecule.

**B**, Region including Lys139 and Lys13 with a tartrate molecule bound between the two lysine side chains.

**C**, $Mg^{2+}$-free active site. An ethylene glycol molecule used as the cryo-protectant was observed.

**D**, $Mg^{2+}$-bound active site.

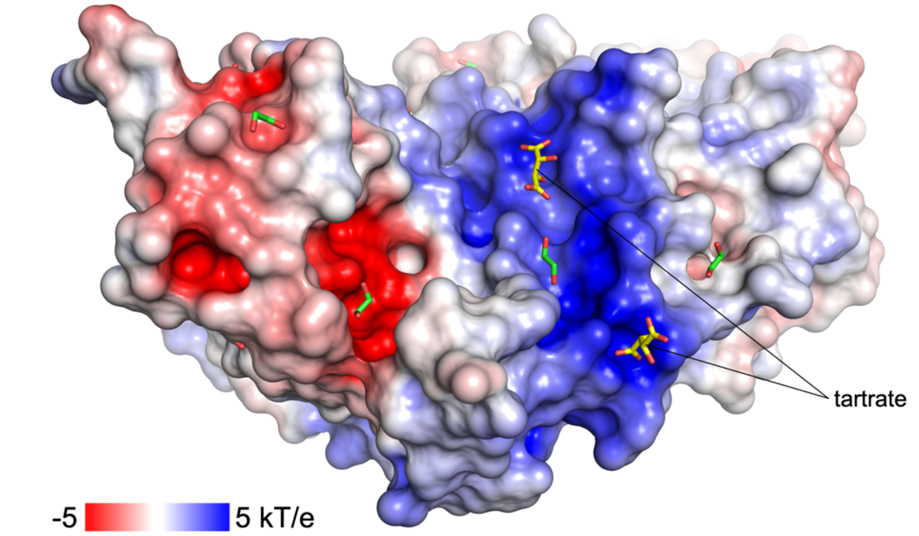


**Fig. S5. Tartrate ions bound on the basic patch of ExoN-nsp10 complex**

Electrostatic surface potential of ExoN-nsp10 with tartrate or ethylene glycol bound on the protein surface.

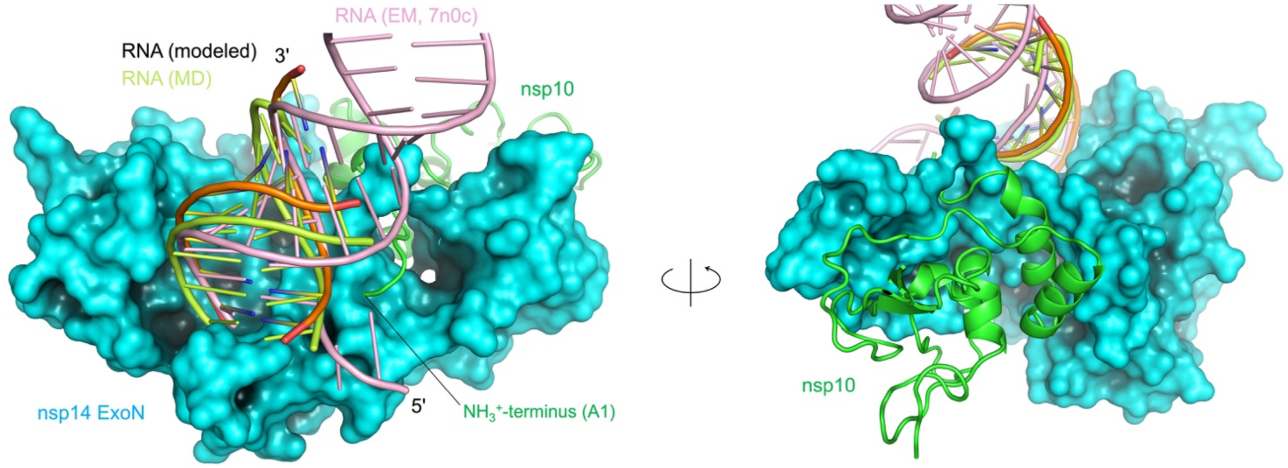


**Fig. S6. ExoN-nsp10-RNA complex models and a cryo-EM structure**

The modeled double-stranded RNA before and after MD simulations are shown in orange and lime, respectively, whereas RNA from a cryo-EM structure (PDB ID: 7N0C) (2) is shown in light pink.

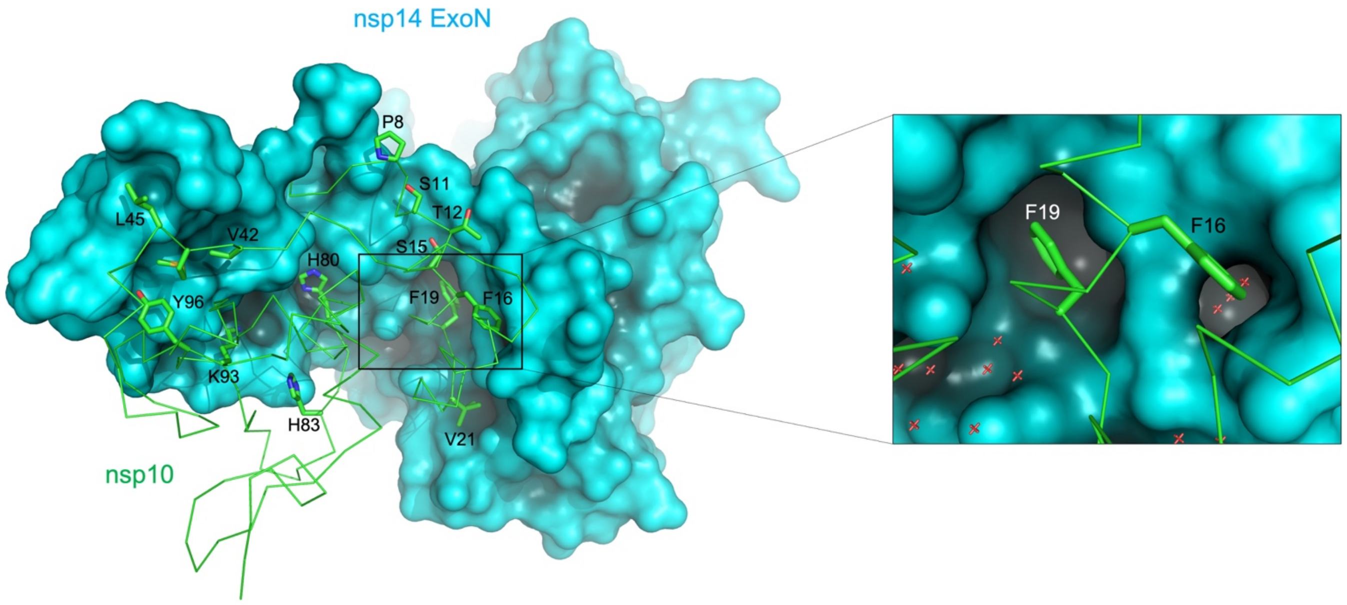


**Fig. S7. ExoN-nsp10 interface**

ExoN domain of nsp14 and nsp10 are shown in solid surface (cyan) and wire-frame (green) representations, respectively. Some of the nsp10 side chains involved in the protein-protein interaction are shown as sticks. A zoomed view of the hydrophobic pocket that accommodates Phe16 and Phe19 of nsp10 is shown on the right. Red crosshairs represent water molecules.

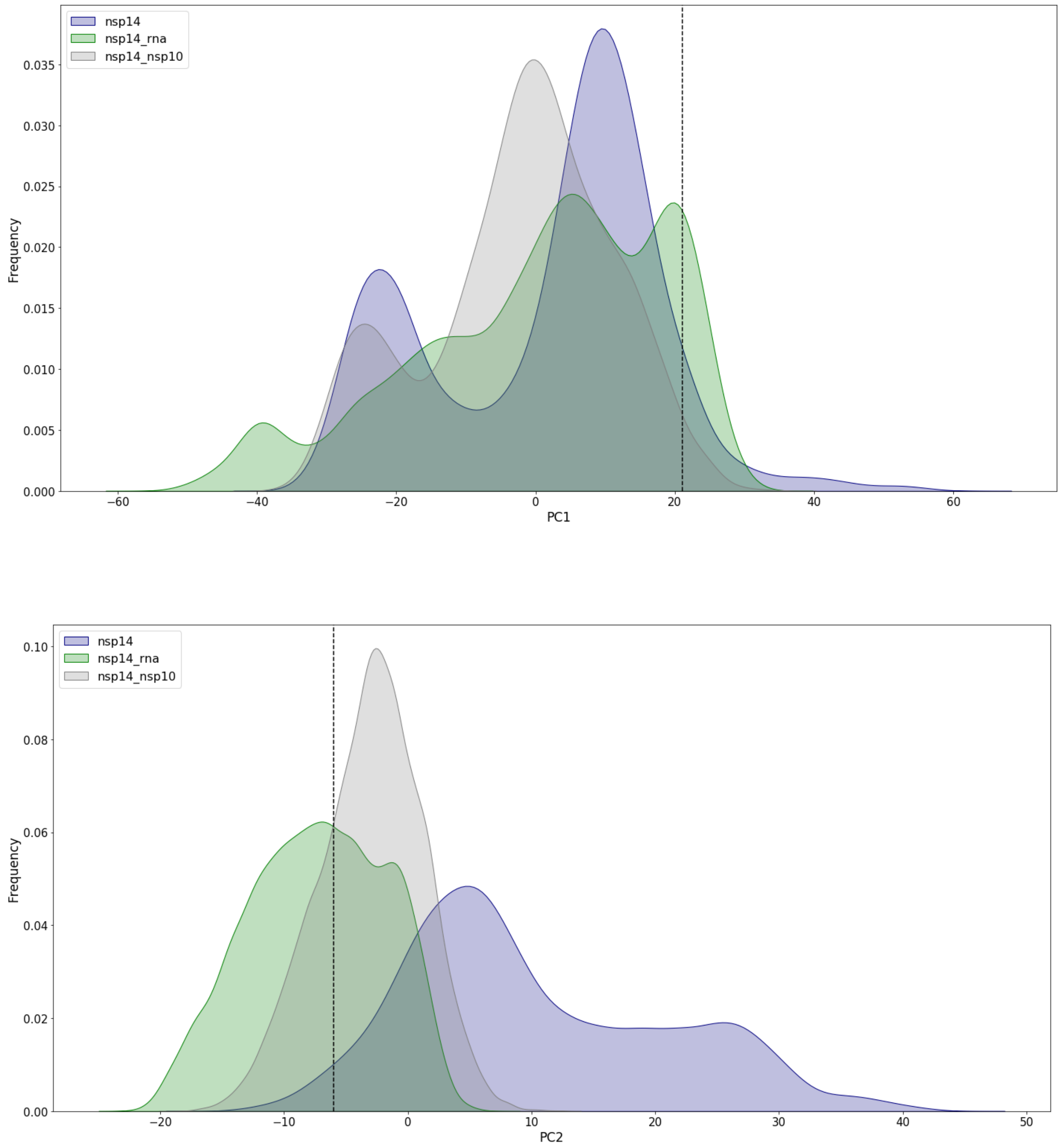


**Fig. S8. Distribution of principal components 1 and 2 (PC1 and PC2) observed in MD simulations.**

The dashed line in each plot indicates the value calculated for the starting structure.

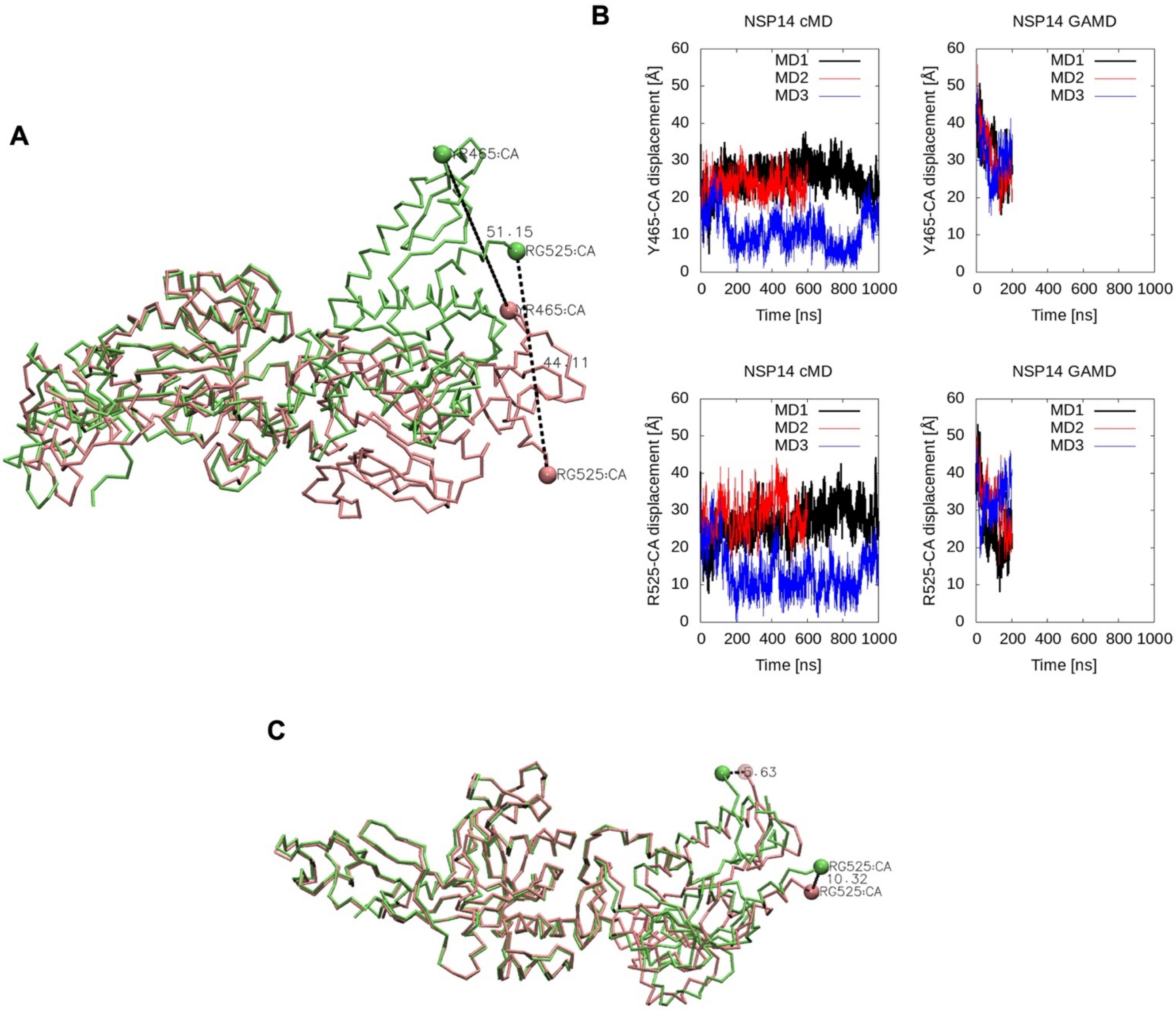


**Fig. S9. Conformational flexibility of full-length nsp14.**

**A**, Superposition of the structures that correspond to PC1 minimum and PC1 maximum values in MD simulations based on the ExoN domain, showing large displacement of the N7-MTase domain (This conformational change is also shown in **Supplementary animation 1**). **B**, Time series showing the displacement of Tyr465 Cα and Arg525 Cα atoms of nsp14 with respect to the MD simulation frame that corresponds to nsp14 PC1 minimum. Displacements are calculated after all frames are superimposed based on the Exon domain (residues 72-290) Cα atoms. **C**, Similar conformational variability, albeit with a smaller magnitude, observed between chains A and B of SARS-CoV nsp14 crystal structure (PDB ID: 5NFY) (3).

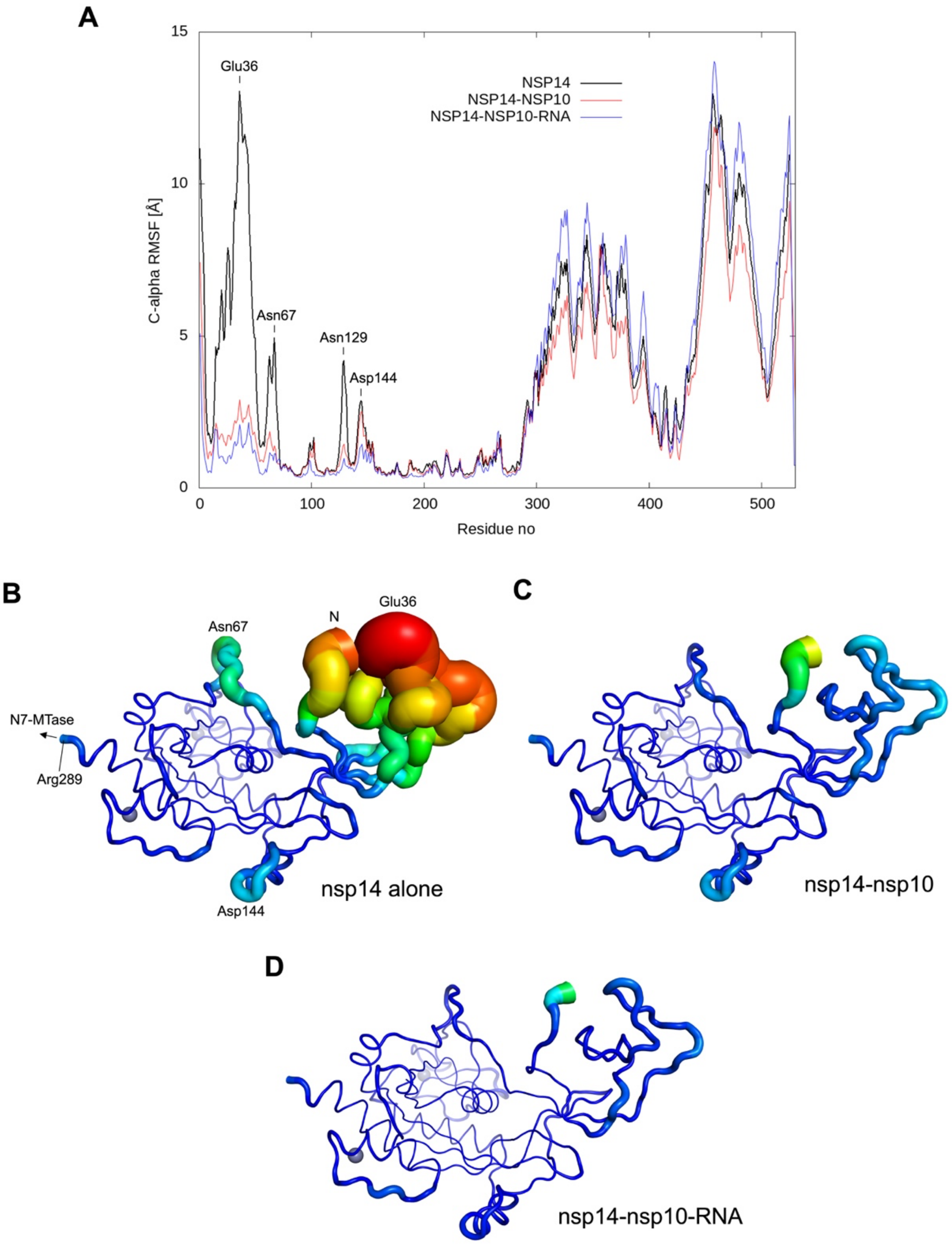


**Fig. S10. Internal dynamics of ExoN domain observed in MD simulations.**

**A**, Root-mean-square fluctuations (RMSF) for nsp14 Cα atoms in MD simulations of the three systems after aligning their trajectories to the starting structure with respect to Cα atoms of nsp14 residues 71-289. **B-D**, RMSF for nsp14 alone (B), nsp14-nsp10 (C), and nsp14-nsp10-RNA (D), depicted by tube

thickness and color. Panels **B** and **C** are same as **Fig. 6 D** and **E**, respectively, and panels **B-D** correspond to the 3 frames in **Supplementary animation 3**.

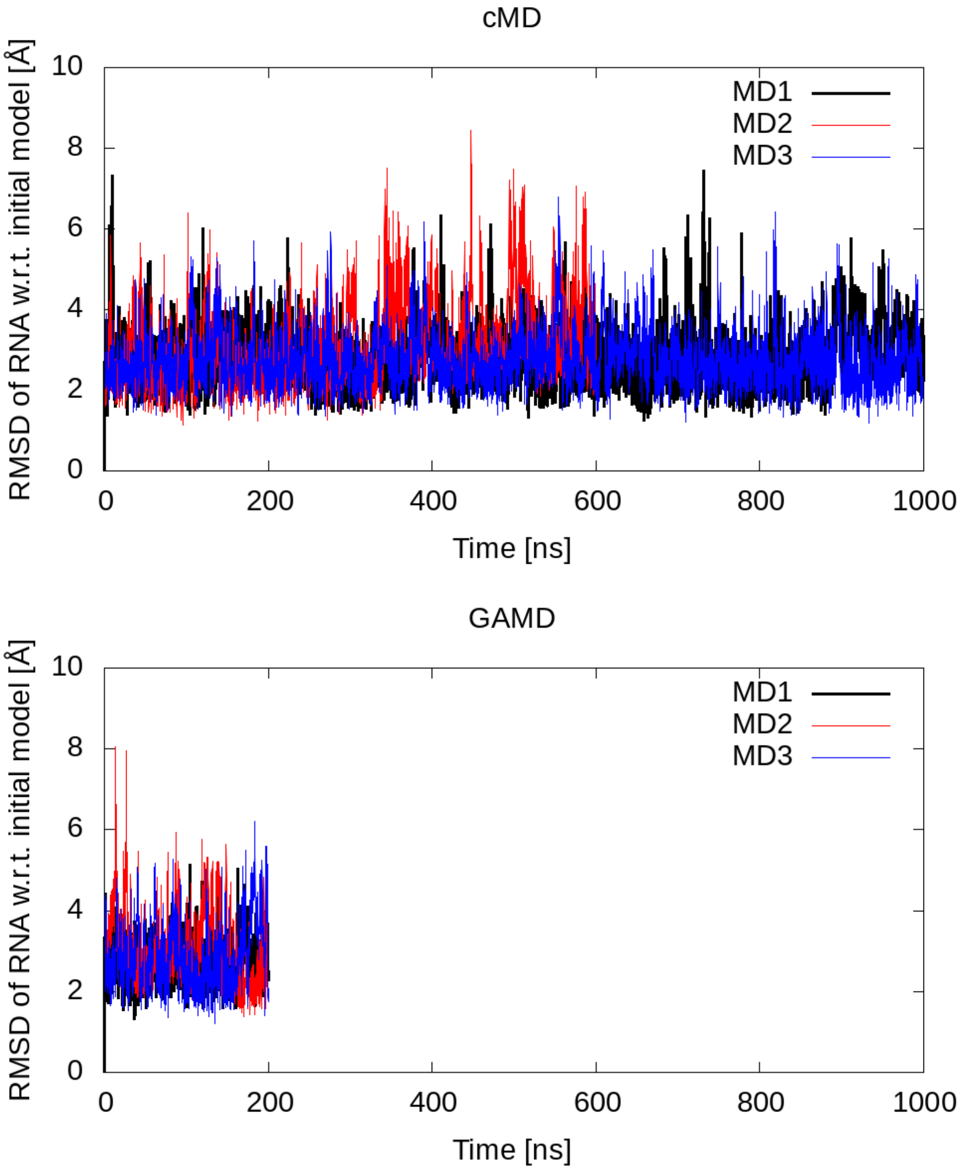


**Fig. S11. Stability of RNA in MD simulations.**

Root-mean-square deviation (RMSD) of RNA atoms calculated for conventional and Gaussian-accelerated MD (cMD and GAMD) simulations after aligning the trajectories with respect to Cα atoms of nsp14 residues 71-289.

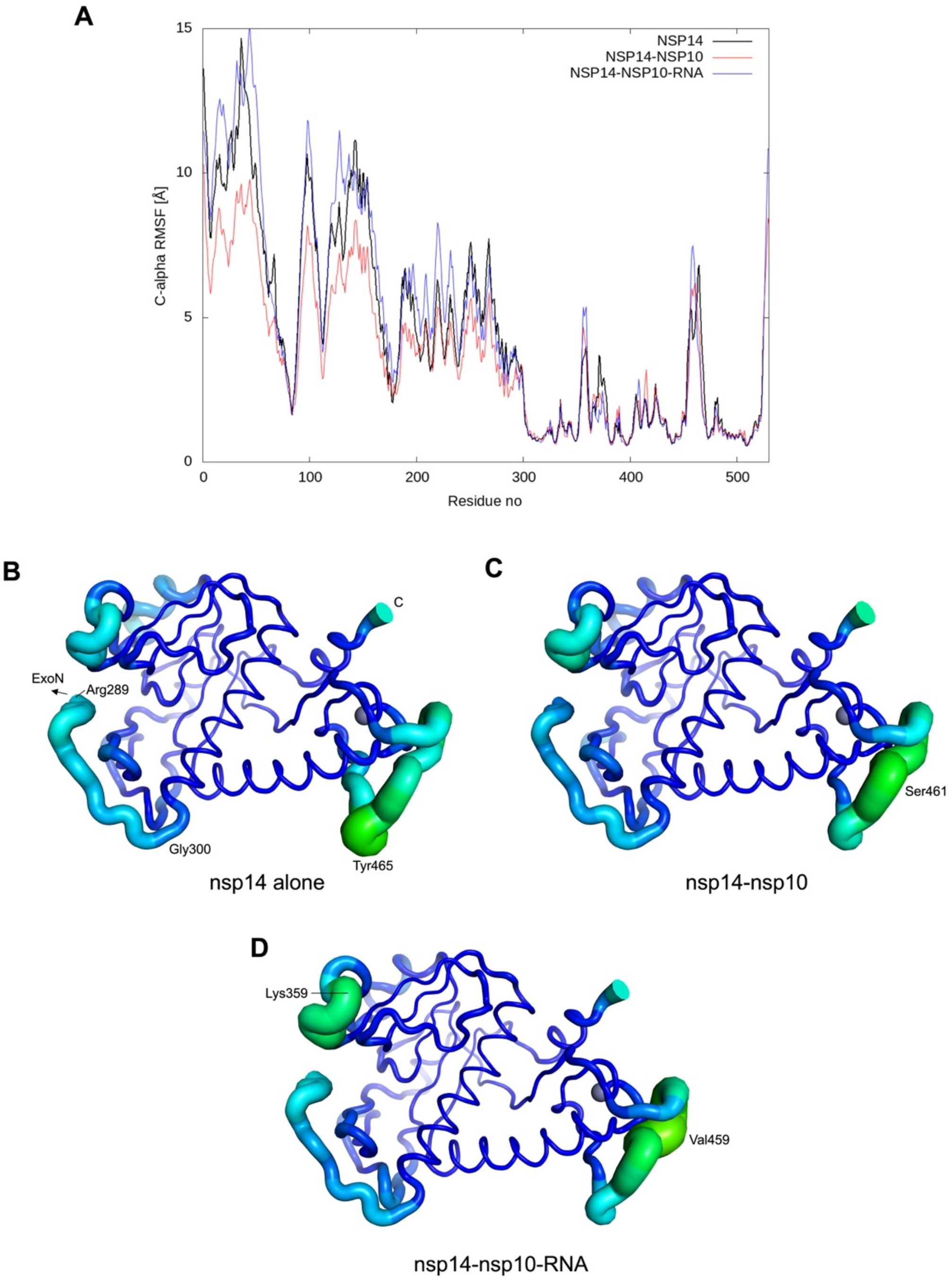


**Fig. S12. Internal dynamics of N7-MTase domain observed in MD simulations.**

**A**, Root-mean-square fluctuations (RMSF) for nsp14 Cα atoms in MD simulations of the three systems after aligning their trajectories to the starting structure with respect to Cα atoms of nsp14 residues 300-525. **B-D**, RMSF for nsp14 alone (B), nsp14-nsp10 (C), and nsp14-nsp10-RNA (D), depicted by tube thickness and color.

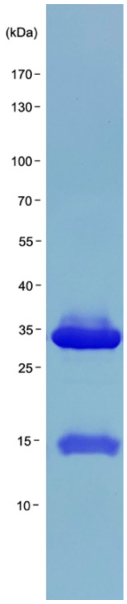


**Fig. S13. SDS-PAGE of purified SARS-CoV-2 ExoN(E191Q)-nsp10 complex.**

This protein complex was used in the crystallographic studies.

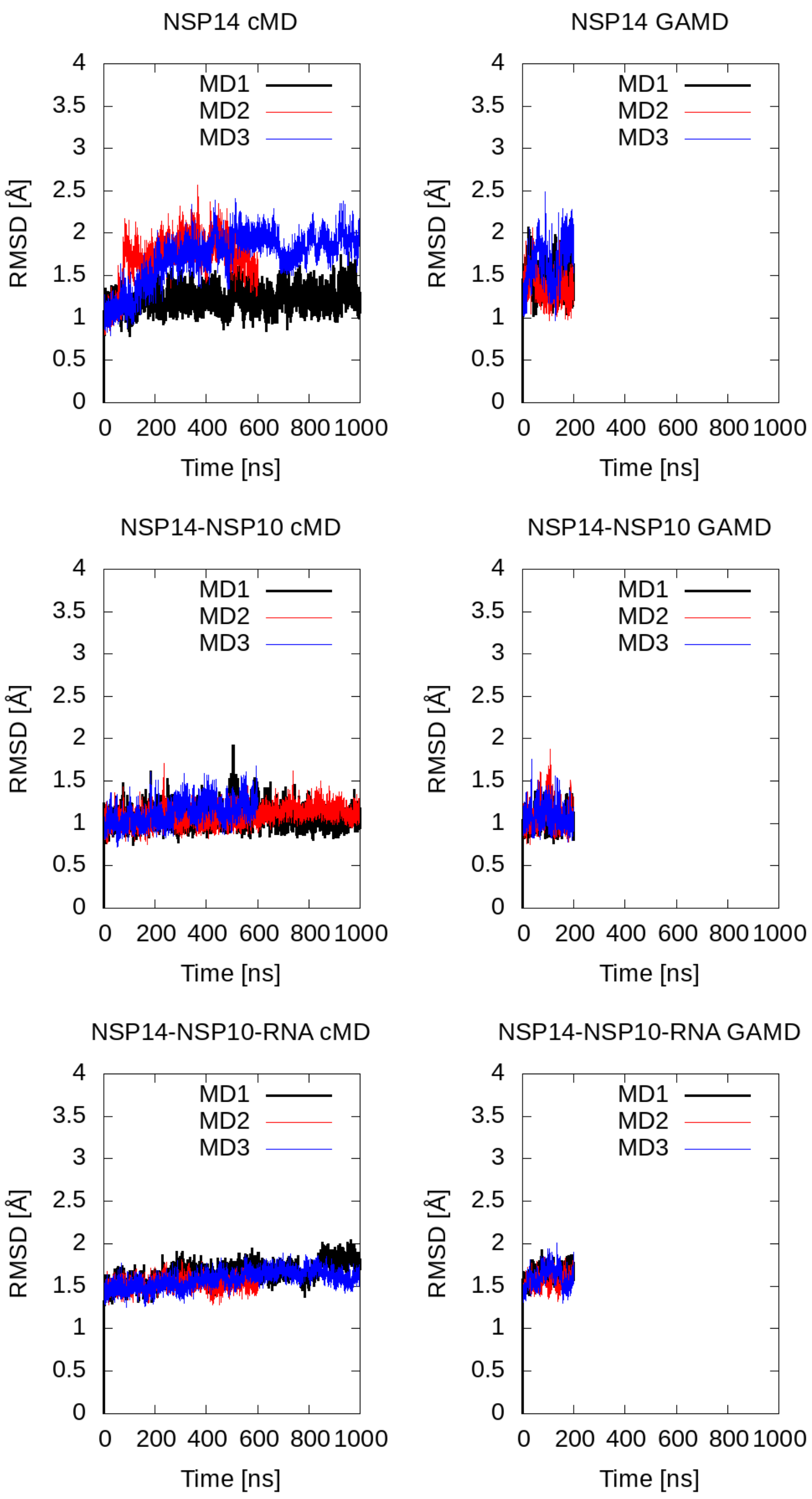


**Fig. S14. Stability of MD simulations.**

Root-mean-square deviation (RMSD) of the nsp14 ExoN domain Cα atoms (residues 71-289) with respect to the initial model throughout MD simulations.

**Supplementary animations (animated gif) legend**

**Supplementary animation 1**

Two nsp14 conformations corresponding to the minimum and maximum of principal component 1 **(**PC1) in MD simulations. The two frames are same as those in **Fig. 6B**.

**Supplementary animation 2**

Two nsp14 conformations corresponding to the minimum and maximum of principal component 2 **(**PC2) in MD simulations. The two frames are same as those in **Fig. 6C**.

**Supplementary animation 3**

Root-mean-square fluctuations (RMSF) for nsp14 ExoN domain C$\alpha$ atoms in MD simulations of the three systems (nsp14 alone, nsp14-nsp10, and nsp14-nsp10-RNA) after aligning their trajectories to the starting structure with respect to C$\alpha$ atoms of nsp14 residues 71-289. RMSF is depicted by varying tube thickness and color. The 3 frames correspond to ***SI Appendix*****, Fig. S10 B-D.**

**SI References**


1. Ma Y., Wu L., Shaw N., Gao Y., Wang J., Sun Y., Lou Z., Yan L., Zhang R., Rao Z. Structural basis and functional analysis of the SARS coronavirus nsp14-nsp10 complex. *Proc. Natl. Acad. Sci. U.S.A.* 112(30):9436-9441 (2015)

2. Liu C., Shi W., Becker S.T., Schatz D.G., Bin L., Yang Y. Structural basis of mismatch recognition by a SARS-CoV-2 proofreading enzyme. *Science* 373(6559):1142-1146 (2021)

3. Ferron F., Subissi L., De Morais A.T.S., Le N.T.T., Sevajol M., Gluais L., Decroly E., Vonrhein C., Bricogne G., Canard B., Imbert I. Structural and molecular basis of mismatch correction and ribavirin excision from coronavirus RNA. *Proc. Natl. Acad. Sci. U.S.A.* 115(2):E162-E171 (2018)
