# Supplementary figures and images for: Structure and dynamics of SARS-CoV-2 proofreading exoribonuclease ExoN
Source: Proc Natl Acad Sci U S A. 2022 Feb 14;119(9):e2106379119. doi: 10.1073/pnas.2106379119 (PMC8892293; doi:10.1073/pnas.2106379119)

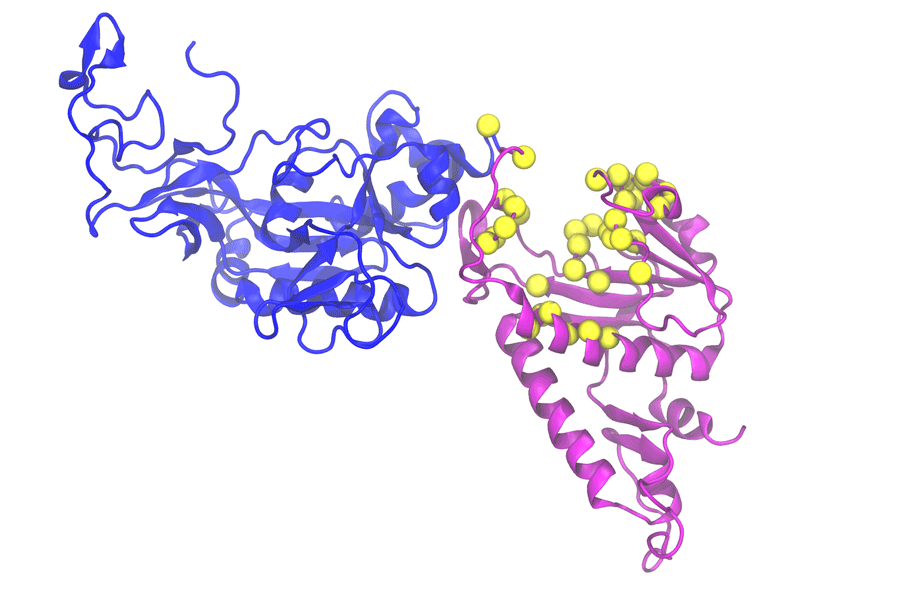

Supplement: Supplementary File [file pnas.2106379119.s01.gif]

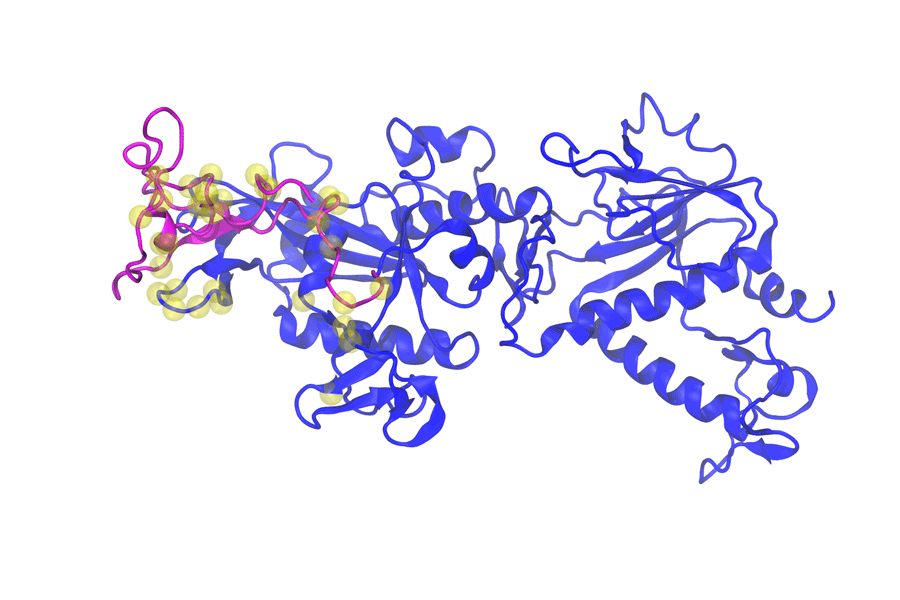

Supplement: Supplementary File [file pnas.2106379119.s02.gif]

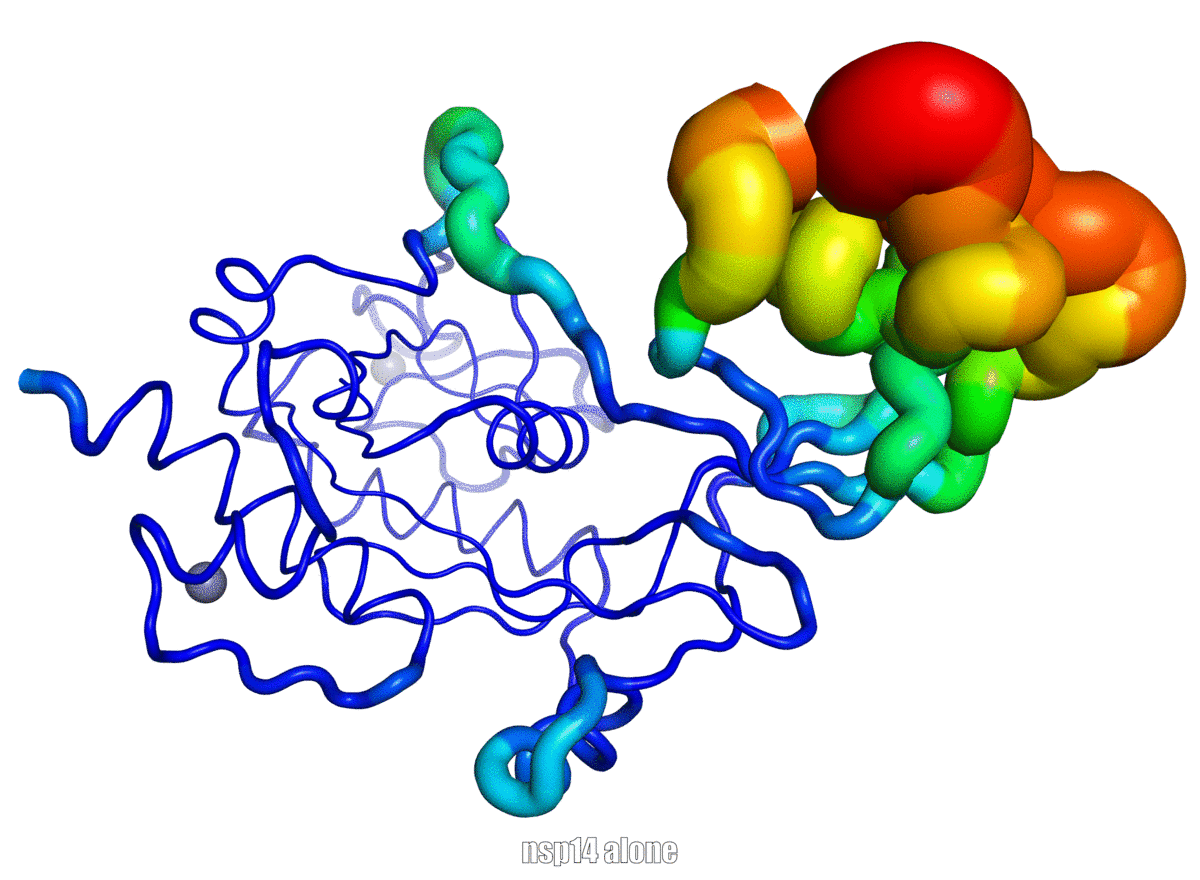

Supplement: Supplementary File [file pnas.2106379119.s03.gif]
